# Bacterial production and direct functional screening of expanded molecular libraries for discovering inhibitors of protein aggregation

**DOI:** 10.1126/sciadv.aax5108

**Published:** 2019-10-16

**Authors:** Dafni C. Delivoria, Sean Chia, Johnny Habchi, Michele Perni, Ilias Matis, Nikoletta Papaevgeniou, Martin Reczko, Niki Chondrogianni, Christopher M. Dobson, Michele Vendruscolo, Georgios Skretas

**Affiliations:** 1 Institute of Chemical Biology, National Hellenic Research Foundation, Athens 11635, Greece.; 2 School of Chemical Engineering, National Technical University of Athens, Athens 15780, Greece.; 3 Centre for Misfolding Diseases, Department of Chemistry, University of Cambridge, Cambridge CB2 1EW, UK.; 4 Faculty of Biology and Pharmacy, Institute of Nutrition, Friedrich Schiller University of Jena, Jena 07743, Germany.; 5 Institute for Fundamental Biomedical Science, Biomedical Sciences Research Center “Alexander Fleming,” Athens 16672, Greece.

## Abstract

Protein misfolding and aggregation are associated with a many human disorders, including Alzheimer’s and Parkinson’s diseases. Toward increasing the effectiveness of early-stage drug discovery for these conditions, we report a bacterial platform that enables the biosynthesis of molecular libraries with expanded diversities and their direct functional screening for discovering protein aggregation inhibitors. We illustrate this approach by performing, what is to our knowledge, the largest functional screen of small-size molecular entities described to date. We generated a combinatorial library of ~200 million drug-like, cyclic peptides and rapidly screened it for aggregation inhibitors against the amyloid-β peptide (Aβ42), linked to Alzheimer’s disease. Through this procedure, we identified more than 400 macrocyclic compounds that efficiently reduce Aβ42 aggregation and toxicity in vitro and in vivo. Finally, we applied a combination of deep sequencing and mutagenesis analyses to demonstrate how this system can rapidly determine structure-activity relationships and define consensus motifs required for bioactivity.

## Teaser

Engineered bacteria enable the screening of vast molecular libraries for discovering inhibitors of pathogenic protein aggregation.

## INTRODUCTION

The phenomenon of protein misfolding and aggregation is a defining feature of a wide range of human diseases with very high socioeconomic impact, including neurodegenerative disorders, type 2 diabetes, and cancer (*1*). Since aggregated proteins can cause disease, either because they can no longer efficiently perform their physiological function (loss of function) or because they form harmful aggregated species with cytotoxic properties (toxic gain of function) (*1*), compounds that prevent, delay, or reverse protein aggregation constitute valuable leads for the development of potential therapeutics. Μany such molecules are currently in preclinical and clinical development (*2*). As a proof of concept for the therapeutic value of this approach, tafamidis, a small molecule that prevents the misfolding and aggregation of the carrier protein transthyretin by binding and stabilizing its tetrameric native form, has been approved for the treatment of familial amyloid polyneuropathy in Europe and Japan (Vyndaqel, Pfizer) (*3*). More recently, migalastat, a chemical rescuer of the misfolding of the lysosomal enzyme α-galactosidase (*4*), has been approved for the treatment of the lysosomal storage disorder Fabry disease in Europe and the United States (Galafold, Amicus Therapeutics). Despite these encouraging results, the vast majority of protein-misfolding diseases remain incurable, as no disease-modifying drug has reached the clinic in most cases. Among the reasons for the failure of current clinical trials, we mention an incomplete understanding of the exact molecular mechanism of action of the anti–amyloid-β peptide (Aβ) candidates and the late treatment of the patients (*5*). Thus, it is imperative to develop systematic and robust approaches to discover previously unidentified and effective disease-modifying agents, which are urgently required for this type of disorders.

Advances in key scientific and technological areas are needed to increase the success rate with which effective drugs against these complex diseases are discovered. One such area is chemical library construction. The availability of molecular libraries with expanded diversities is expected to markedly increase the chances for identifying compounds with the desired properties (*6*, *7*). Because of current limitations in organic synthesis and the isolation of natural products, however, the diversity of currently tested small-molecule libraries is typically not higher than 10^5^ to 10^6^ (*8*). Considering that the size of the chemical space for small molecules, i.e., the number of all possible low–molecular weight structures has been estimated to be ~10^60^ (*9*), it is clear that drug screening efforts will benefit from increased diversity. In addition, even when chemical libraries with larger sizes are available, the majority of screening methodologies for the identification of drug-like compounds are not sufficiently high throughput to efficiently handle very large libraries. Functional screening assays in multiwell plate format, for example, become impractical for libraries with more than 10^6^ to 10^7^ members.

Genetically encoded combinatorial libraries can enable a marked expansion in the number and chemical complexity of lower–molecular weight compounds, which can be generated and subsequently tested for bioactivity (*7*, *10*, *11*). By using approaches of this type, molecular libraries with diversities ranging from many millions to even tens of trillions of test compounds have already been generated (*6*, *7*, *10*, *12*, *13*), and molecules with valuable biological activities have been discovered. These bioactivities include modulation of the aggregation process of misfolding-prone and disease-associated proteins, such as the Aβ and huntingtin (*11*, *14*–*17*).

One important shortcoming when investigating DNA-encoded libraries for protein misfolding and aggregation diseases, however, is that they can only be screened for binding against immobilized protein targets (*10*). Despite their efficiency in identifying strong binders, these affinity-based selections cannot readily provide functional information regarding the aggregation inhibition activity of the identified hits (*10*). As a result, the selected binders need to be resynthesized chemically and evaluated again for aggregation-inhibitory activity in secondary assays. This procedure adds substantial time, complexity, and cost to the overall screening process and is regarded as a major bottleneck by the pharmaceutical industry (*12*). Furthermore, in many cases, the outcome of the selection process results in the identification of a large fraction of hits that are either completely inactive (*12*) or have opposite effects on protein misfolding and aggregation than the ones intended originally (*15*).

In an effort to generate new and efficient systems for discovering previously unidentified inhibitors of pathogenic protein aggregation, we have recently reported the development of a synthetic biology platform that enables the discovery of chemical rescuers of disease-associated protein misfolding (*18*). In this system, combinatorial libraries of lower–molecular weight peptide macrocycles are biosynthesized in *Escherichia coli* cells and are simultaneously screened for their ability to correct the problematic folding of misfolding-prone, disease-associated proteins using a flow cytometric ultrahigh-throughput genetic screen.

In the present work, we demonstrate how this bacterial discovery platform can be expanded to enable the production and direct functional screening of molecular libraries with greatly increased diversities, thus considerably surpassing the capabilities of other systems reported to date. We used this system to generate a combinatorial library of ~200 million peptide macrocycles and to perform simultaneous functional screening for aggregation inhibition activity against the 42-residue form of Αβ (Αβ42), which is associated with Alzheimer’s disease. Within a matter of only a few days, our bacterial platform enabled the production and screening of the complete library and identified hundreds of hits. Analysis of the selected macrocycles revealed that they form different clusters with distinct sequence characteristics. Selected macrocycles derived from the most dominant clusters were subjected to in vitro biochemical and biophysical testing and were found to be highly potent inhibitors of Aβ42 aggregation at substoichiometric ratios. In vivo testing in established models of Alzheimer’s disease in the nematode *Caenorhabditis elegans* demonstrated that the selected macrocycles were effective in decreasing the deposition of Aβ42 aggregates and in markedly reversing Aβ42-induced pathogenic effects. We then used a combination of high-throughput sequencing and site-directed mutagenesis analyses to determine structure-activity relationships for the selected macrocycles and to define consensus motifs required for high bioactivity in these molecules. Overall, our discovery platform enables the simultaneous production and functional screening of molecular libraries with markedly expanded diversities for the identification of compounds with therapeutic potential for inhibiting the aggregation of disease-associated polypeptides.

## RESULTS

### Construction and characterization of a low-weight molecular library with expanded diversity

The molecular libraries that we have chosen to use for the discovery of protein aggregation inhibitors are combinatorial libraries of head-to-tail cyclic heptapeptides, with an average molecular mass of about 770 Da. These macrocycles fall within the class of small molecules (molecular mass, <900 Da) but occupy an area of chemical space beyond the classical Lipinski’s rule of five (bRo5 space; molecular mass, 500 to 1000 Da), where different rules for drug-likeness compared to classical small-molecule therapeutics apply (*19*, *20*). The very large number of possible amino acid combinations comprising a peptide sequence (of seven amino acids in our case) enables greatly expanded levels of molecular diversity compared to available synthetic and natural small-molecule libraries (*8*). Furthermore, the cyclic nature of these molecules affords higher binding affinities for other proteins, enhanced ability to penetrate biological barriers, and enhanced resistance to proteolysis compared to their linear analogs (*21*).

**Fig. 1:**
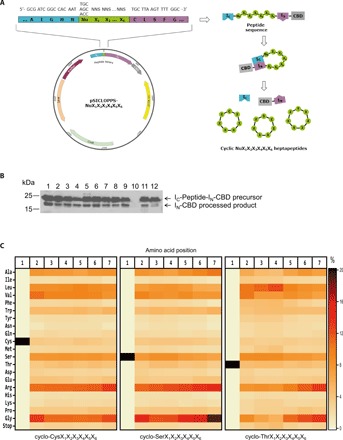
Construction and characterization of a combinatorial cyclic heptapeptide library with expanded diversity. (**A**) Left: Representation of the pSICLOPPS-NuX_1_X_2_X_3_X_4_X_5_X_6_ vector library encoding the combinatorial heptapeptide library cyclo-NuX_1_X_2_X_3_X_4_X_5_X_6_. Nu: Cys, Ser, or Thr; X: any of the 20 natural amino acids; NNS: randomized codons, where N = A, T, C, or G and S = G or C; I_C_: C-terminal domain of the Ssp DnaE split intein; I_N_: N-terminal domain of the Ssp DnaE split intein. Right: Peptide cyclization using the SICLOPPS construct. Upon interaction between the two intein domains I_C_ and I_N_, the encoded I_C_-NuX_1_X_2_X_3_X_4_X_5_X_6_-I_N_-CBD fusions undergo intein splicing and peptide cyclization, leading to the production of the cyclo-NuX_1_X_2_X_3_X_4_X_5_X_6_ library. (**B**) Western blot analysis of 12 randomly picked individual clones from the combinatorial heptapeptide library cyclo-NuX_1_X_2_X_3_X_4_X_5_X_6_, showing the expression and processing of the precursor fusion protein I_C_-peptide-I_N_-CBD. The 25-kDa band corresponds to the unprocessed precursor and the 20-kDa band to the processed I_N_-CBD construct, and indicates, wherever present, successful intein splicing and peptide cyclization. Clone 10, for which the precursor is not expressed, was to contain a stop codon in its peptide-encoding region. (**C**) Heatmap representation of the amino acid distribution at each position of the constructed cyclo-CysX_1_X_2_X_3_X_4_X_5_X_6_ (left), cyclo-SerX_1_X_2_X_3_X_4_X_5_X_6_ (middle), and cyclo-ThrX_1_X_2_X_3_X_4_X_5_X_6_ (right) sublibraries, as demonstrated by the deep sequencing analysis results.

Libraries of head-to-tail cyclic peptides can be conveniently produced in *E. coli* cells by the “split intein–mediated circular ligation of peptides and proteins” (SICLOPPS) method, where a circularly permuted split intein catalyzes the formation of a peptide bond between the termini of the target protein or peptide (*22*). SICLOPPS is a well-established technique, which has been previously used to identify cyclic peptides with different bioactivities (*23*). The only external requirement for the intein splicing reaction and peptide cyclization to take place is the presence of a nucleophilic amino acid (Cys, Ser, or Thr) as the first amino acid of the to-be-cyclized peptide (*18*). Thus, to maximize the diversity of our macrocycle library, we constructed a combinatorial heptapeptide library with the general formula cyclo-NuX_1_X_2_X_3_X_4_X_5_X_6_, where Nu is any one of the nucleophilic amino acids Cys, Ser, or Thr and X is any one of the 20 natural amino acids. The maximum theoretical diversity of this library is 3 × 20^6^ = 192 million different sequences. The libraries of genes encoding these cyclic heptapeptide libraries were constructed using degenerate polymerase chain reaction (PCR) primers, in which the randomized amino acids (X) were encoded using randomized NNS codons, where N is A, T, G, or C and S is G or C (see Materials and Methods). The generated peptide-encoding gene libraries were cloned into the vector pSICLOPPS (*18*) to form the combined pSICLOPPS-NuX_1_X_2_X_3_X_4_X_5_X_6_ vector library (Fig. 1A). These vectors express a combinatorial library of tetrapartite fusion proteins comprising the following: (i) the C-terminal domain of the Ssp DnaE intein (I_C_), (ii) a NuX_1_X_2_X_3_X_4_X_5_X_6_ heptapeptide sequence, (iii) the N-terminal domain of the Ssp DnaE intein (I_N_), and (iv) a chitin-binding domain (CBD) for immunodetection and/or purification, under the control of the P_BAD_ promoter and its inducer l(+)-arabinose (Fig. 1A). Cloning of the resulting gene libraries into the pSICLOPPS plasmid yielded a total of 1.2 × 10^9^ independent transformants, as judged by plating experiments after serial dilutions.

To assess the quality of our constructed library, we initially chose 150 randomly selected clones and performed colony PCR and SDS–polyacrylamide gel electrophoresis (SDS-PAGE)/Western blot. This analysis revealed that approximately 45% of the analyzed clones contained a DNA insert of the correct size and produced full-length I_C_-peptide-I_N_-CBD precursor fusion protein (molecular mass, ~25 kDa), which could undergo processing (appearance of a band with a molecular mass of ~20 kDa) (Fig. 1B). Τhis processing is a prerequisite for successful intein splicing and indicates possible formation of a cyclic product. According to these results, the generated library contains approximately 5.6 × 10^8^ clones that apparently produce cyclic heptapeptides, a number that covers the theoretical diversity of our combined library by almost threefold.

To characterize the constructed library further, we performed deep sequencing analysis of the peptide-encoding region of the pSICLOPPS-NuX_1_X_2_X_3_X_4_X_5_X_6_ vector library. Of the ~3.4 million plasmid sequences that we analyzed, ~75% were unique at the DNA level and ~95% of those were found to encode unique peptide sequences (table S1). All amino acids were found to be encoded at every position of the generated library, albeit with an overrepresentation of residues corresponding to Gly and Arg (Fig. 1C). Together, these results indicate that we have constructed a very high-diversity library encoding the vast majority, if not all, of the theoretically possible ~192 million cyclo-NuX_1_X_2_X_3_X_4_X_5_X_6_ heptapeptide sequences.

### Ultrahigh-throughput functional screening for Αβ42 aggregation inhibitors

**Fig. 2:**
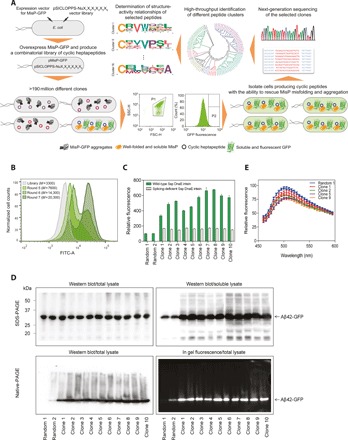
Biosynthesis and ultrahigh-throughput screening of a combinatorial cyclic heptapeptide library with expanded diversity for discovering inhibitors of protein aggregation. (**A**) Schematic of the used bacterial platform for discovering inhibitors of protein aggregation and for the high-throughput analysis of the selected hits. pMisP-GFP: plasmid encoding a misfolded protein-GFP fusion; pSICLOPPS-NuX_1_X_2_X_3_X_4_X_5_X_6_: vector library encoding the combinatorial heptapeptide library cyclo-NuX_1_X_2_X_3_X_4_X_5_X_6_; Nu: Cys, Ser, or Thr; X:, any of the 20 natural amino acids; FSC-H: forward scatter; SSC-H: side scatter; P: sorting gate. (**B**) FACS of *E. coli* Tuner (DE3) cells overexpressing Aβ42-GFP and the combined cyclic heptapeptide library. *M*: mean GFP fluorescence in arbitrary units. FITC-A: filter for fluorescein isothiocyanate. (**C**) Relative fluorescence of *E. coli* Tuner (DE3) cells overexpressing Aβ42-GFP and 10 randomly selected cyclic heptapeptide clones isolated after the seventh round of FACS shown in (B) and using either the wild-type split Ssp DnaE intein (green bars) or the splicing-deficient variant H24L/F26A (white bars) (*25*). Two randomly picked cyclic peptide sequences (random 1 and 2) previously shown to have no effect on Αβ42-GFP fluorescence and aggregation (*18*) were used as a negative control. The fluorescence of the bacterial population producing cyclic peptide random 1 was arbitrarily set to 100. Mean values ± SEM are presented (*n* = 3 independent experiments, each performed in three replicates). (**D**) Top: Western blot analysis of total (left) and soluble (right) lysates of *E. coli* Tuner (DE3) cells overexpressing Aβ42-GFP and the 10 individual cyclic peptide sequences tested in (C). The predicted molecular mass of the Aβ42-GFP fusion is ~32 kDa. Bottom: Western blotting using the anti-Aβ antibody 6E10 (left) and in-gel fluorescence (right) analyses of total lysates following native PAGE of *E. coli* Tuner (DE3) cells coexpressing Aβ42-GFP and the 10 individual cyclic peptide sequences tested in (C). (**E**) Emission spectra of *E. coli* Tuner (DE3) cells overexpressing Aβ42 along with four of the selected cyclic heptapeptide sequences tested in (C) and stained with ThS. The maximum fluorescence of the bacterial population producing cyclic peptide random 1 was arbitrarily set to 100. Mean values ± SEM are presented (*n* = 1 experiment performed in three replicates).

To perform direct functional screening of our vast library of cyclic heptapeptides and readily identify bioactive macrocyclic inhibitors of pathogenic protein aggregation, we used an ultrahigh-throughput system that we previously developed (*18*). Because of the high aggregation propensity of Aβ, *E. coli* cells overexpressing Aβ42–green fluorescent protein (GFP) produce a misfolded fusion that eventually accumulates into insoluble inclusion bodies lacking fluorescence (*24*). Conditions that inhibit Aβ aggregation, however, result in the formation of soluble and fluorescent Aβ42-GFP, and bacterial cells expressing this fusion acquire a fluorescent phenotype (*18*, *24*). On the basis of this, production of the macrocyclic peptide libraries under investigation and their screening for misfolding-rescuing activity in this system are carried out simultaneously in *E. coli* cells in an integrated fashion, by selecting and isolating the bacterial clones biosynthesizing the molecules that enhance the fluorescence of chimeric fusions of misfolding-prone proteins with the GFP (Fig. 2A).

Electrocompetent *E. coli* Tuner (DE3) cells carrying the expression vector pETAβ42-GFP (*24*), which produces Aβ42-GFP under the control of the strong bacteriophage T7 promoter, were cotransformed with the pSICLOPPS-NuX_1_X_2_X_3_X_4_X_5_X_6_ vector library. Approximately 3 × 10^9^ transformants carrying both vectors were harvested, pooled together, and grown in Luria-Bertani (LB) liquid medium containing 0.005% l(+)-arabinose—the inducer of cyclic peptide production—at 37°C with shaking. When the optical density at 600 nm (OD_600_) of the bacterial culture reached a level of about 0.5, 0.1 mM isopropyl-β-d-thiogalactoside (IPTG) was added to the medium so as to induce overexpression of the Aβ42-GFP reporter. After about 2 hours at 37°C, ~3 × 10^9^ cells were screened, and the population exhibiting the top 1 to 3% fluorescence was isolated using fluorescence-activated cell sorting (FACS) (fig. S1A). The isolated cells were regrown and screened for a total of seven rounds, at which point the mean fluorescence of the population displayed an almost sixfold increase compared to the starting library (Fig. 2B). No further substantial increase in fluorescence was observed after additional rounds of sorting.

After the seventh round of FACS screening, 10 individual clones were randomly chosen from the sorted population, and their peptide-encoding vectors were isolated and then retransformed into fresh *E. coli* Tuner (DE3) cells carrying pET Aβ42-GFP. Protein production was induced from both plasmids, and the levels of Aβ42-GFP fluorescence of these cultures were measured. Aβ42-GFP fluorescence of the isolated peptide-expressing clones was found to be markedly increased compared to cells expressing the same Aβ42-GFP fusion in the presence of two random cyclic peptide sequences previously found to have no effect on Aβ42-GFP fluorescence and aggregation (Fig. 2C) (*18*). All isolated clones expressed a full-length intein-peptide fusion (~25 kDa), which could undergo processing to yield a lower–molecular weight band corresponding to excised I_N_-CBD (~20 kDa), thus suggesting successful intein processing and possible formation of a cyclic peptide product (fig. S1B). Furthermore, the observed phenotypic effects were dependent on the ability of the Ssp DnaE intein to perform protein splicing, as the double amino acid substitution H24L/F26A in the C-terminal half of the Ssp DnaE intein, which is known to abolish asparagine cyclization at the I_C_/extein junction and prevent extein splicing and peptide cyclization (*25*), was found to reduce Aβ42-GFP fluorescence back to wild-type levels (Fig. 2C and fig. S1B). Last, the observed increases in fluorescence were found to be Aβ42 specific, as the isolated pSICLOPPS-NuX_1_X_2_X_3_X_4_X_5_X_6_ vectors did not enhance the levels of cellular green fluorescence when the sequence of Aβ42 was replaced in the same vector with that of the DNA-binding (core) domain of human p53 containing a tyrosine to cysteine substitution at position 220 [p53C (Y220C)], a protein whose misfolding and aggregation is associated with certain forms of cancer (fig. S1C) (*26*).

Analysis of the expressed Aβ42-GFP fusions by SDS-PAGE and Western blotting revealed that the bacterial clones expressing the selected cyclic heptapeptides produce markedly increased levels of soluble Aβ42-GFP compared to random cyclic peptides, despite the fact that accumulation of total Aβ42-GFP protein remained at similar levels (Fig. 2D, top, and fig. S1D). Furthermore, when the same cell lysates were analyzed by native PAGE and Western blotting, we observed that coexpression of the selected cyclic peptides reduced the accumulation of higher-order Aβ42-GFP aggregates, which could not enter the gel, and increased the amounts of species with higher electrophoretic mobility (Fig. 2D, bottom left). These higher electrophoretic mobility species correspond to the fraction of the total Aβ42-GFP that exhibits fluorescence (Fig. 2D, bottom right). Since the solubility and fluorescence of bacterially expressed Aβ42-GFP has been found to be inversely proportional to the aggregation propensity of Aβ42 (*18*, *24*, *27*), the results described above suggest that Aβ42 aggregation is markedly decreased in the presence of the selected cyclic heptapeptides. Similar results were acquired when Aβ42 was produced in an unfused, GFP-free form. When we tested the effects of the selected cyclic heptapeptides on Aβ42 aggregation with an in vivo assay using whole-cell staining of intracellular formation of Aβ42 aggregates with thioflavin S (ThS) (*28*), we observed that coproduction of the selected peptides resulted in decreased levels of ThS fluorescence, further indicating a reduced aggregate formation (Fig. 2E).

DNA sequencing of the 10 selected clones revealed five distinct cyclic heptapeptide sequences: cyclo-CKVWQLL (present six times among the sequenced clones), cyclo-CRVWTEL, cyclo-CKVWMPL, cyclo-CIVVPSI, and cyclo-CRIVPSL (fig. S1E).

### High-throughput analysis of the isolated hits

We previously found that low–molecular weight peptide macrocycles are a rich source of chemical rescuers of disease-associated protein misfolding and aggregation (*18*). On the basis of that initial observation, and in combination with the fact that multiple distinct cyclic heptapeptide sequences were identified among the 10 selected clones initially tested (fig. S1E), we hypothesized that numerous Aβ42-targeting macrocyclic sequences may exist among the selected peptide pool. To determine the entire ensemble of potentially bioactive cyclic heptapeptides present in our library, we performed deep sequencing analysis of the heptapeptide-encoding regions in >0.4 million pSICLOPPS-NuX_1_X_2_X_3_X_4_X_5_X_6_ vectors contained in the selected bacterial population after the seventh round of sorting (Fig. 2B). This analysis revealed 416 distinct cyclic heptapeptide sequences appearing at least 20 times within the sorted population, thus indicating that their presence in the selected pool is not coincidental. Cloning of three randomly chosen cyclic heptapeptide sequences appearing in the sorted pool only with very low frequencies revealed that they are also efficient in increasing the fluorescence of bacterially expressed Aβ42-GFP (fig. S1F).

**Fig. 3:**
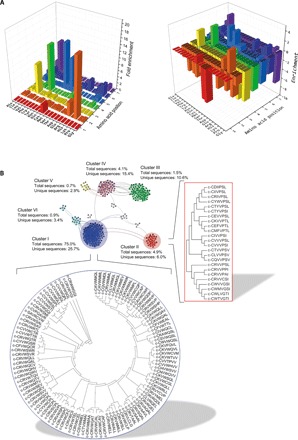
Sequence analysis of the selected cyclic heptapeptide pool. (**A**) Left: Frequency of appearance of the 20 natural amino acids at each position of the heptapeptide sequences selected after the seventh round of sorting (Fig. 2B). Right: Enrichment of the 20 natural amino acids at each position of the heptapeptide sequences selected after the seventh round of sorting (Fig. 2B). Values represent the log_2_-fold change of the amino acid frequency of appearance of the peptides from the sorted pool compared to the initial library. (**B**) Visualization of the main clusters formed by the selected cyclic heptapeptides according to their sequence similarities. Nodes represent different cyclic peptide sequences, and solid lines connect pairs of peptides that share at least 70% sequence identity. The sequences of the members of the two most dominant clusters (clusters I and II) are shown in the corresponding dendrograms.

We next performed sequence analysis of the selected cyclic heptapeptides. We found that Cys was the nucleophilic amino acid that was present at position 1 in the vast majority of the selected cyclic heptapeptides (99.6% of all selected sequences) (Fig. 3A, left). Furthermore, we observed that the frequency of appearance of only a very small number of specific amino acids was enriched at each position among the selected sequences: Arg and Lys at position 2; Val at position 3; Trp and Thr at position 4; Ile, Gln, Cys, Met, Ser, Thr, and Pro at position 5; Ala, Leu, Val, Glu, Lys, and Pro at position 6; and Ile, Leu, and Pro at position 7 (Fig. 3A, right, and table S2). On the contrary, the majority of amino acids, including the ones that were present in higher abundance in the initial library, were strongly de-enriched (Fig. 3A, right, and table S2), thus indicating a highly efficient selection process.

To identify potential relationships among the selected cyclic heptapeptides, we carried out sequence similarity analysis and hierarchical clustering. As the similarity analysis is performed using linear sequences, all possible circular permutations of each selected cyclic heptapeptide were taken into consideration (fig. S2A). From the 416 cyclic heptapeptides selected, 323 of them formed 1467 unique pairs with more than 70% sequence identity and formed 20 distinct clusters with similar sequence characteristics (Fig. 3B and fig. S2B). Clusters I and II were the most dominant, comprising 75.0 and 4.9% of the selected bacterial clones, respectively, as well as 25.7 and 6% of the unique cyclic heptapeptide sequences selected (Fig. 3B and table S3). The majority of peptides from clusters I and II appeared to belong to a cyclo-CxVWxxx and a cyclo-CxxVPSx motif, respectively, in agreement with our previous observations (fig. S1E).

### The selected cyclic heptapeptides inhibit Aβ42 aggregation in vitro

**Fig. 4:**
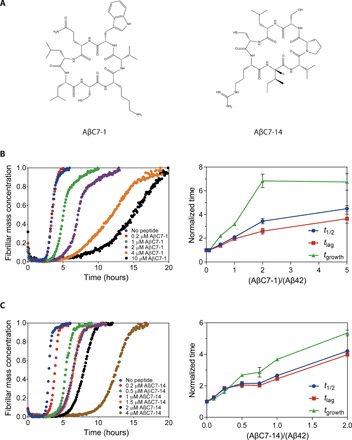
AβC7-1 and AβC7-14 inhibit Aβ42 aggregation in vitro. (**A**) Chemical structures of the selected cyclic heptapeptides AβC7-1 and AβC7-14. (**B**) Kinetic profiles of the aggregation of 2 μM Aβ42 in the absence and presence of AβC7-1 at different molar ratios (left) and the normalized *t*_1/2_, *t*_lag_, and *t*_growth_ values of the corresponding aggregation reactions (right). (**C**) As in (B) for AβC7-14. In (B) and (C), mean values ± SEM are presented (*n* = 1 experiment performed in three replicates).

Two of the selected heptapeptides, cyclo-CKVWQLL and cyclo-CRIVPSL, termed AβC7-1 and AβC7-14 (Aβ-targeting cyclic 7-peptide number 1 and 14), respectively (Fig. 4A and table S4), were chosen for subsequent analysis and were synthetized chemically in milligram quantities (fig. S3A). These cyclic peptides were selected because they were both encountered in the postselection pool investigated initially (fig. S1E) and, more importantly, they were the most frequently encountered members among the two most dominant clusters (clusters I and II) (table S4).

AβC7-1 and AβC7-14 were initially evaluated by monitoring their effects on the kinetics of Aβ42 aggregation by thioflavin T (ThT) staining using a highly reproducible approach previously described (*29*, *30*). Monomeric Aβ42 was purified after recombinant production in *E. coli*, and aggregation kinetic experiments were initiated using 2 μM Aβ42 in the absence and presence of AβC7-1 and AβC7-14. Both AβC7-1 and AβC7-14 inhibited Aβ42 aggregation very effectively at substoichiometric ratios as low as 0.5 molar equivalents for AβC7-1 and 0.1 molar equivalents for AβC7-14 (Fig. 4, B and C). Specifically, we found that both the *t*_lag_ (time required for the ThT fluorescence to reach 10% of the total amplitude) and *t*_growth_ (transition time from 10 to 90% of the total ThT fluorescence amplitude) of the Aβ42 aggregation reaction were increased in the presence of the two selected macrocycles, albeit to a different extent (Fig. 4, B and C, right). Furthermore, we found that the Aβ42 fibrils formed after the completion of the aggregation reaction in the absence and presence of both AβC7-1 and AβC7-14 were similar in both size and morphology (fig. S3B). Thus, it is likely that these selected macrocycles are not binding irreversibly to Aβ42 species and redirecting the aggregation process toward off-pathway aggregates. The observed deceleration of Aβ42 aggregation by the selected macrocycles could also be observed in the absence of ThT, when the progress of the aggregation was monitored by extracting aliquots at different time points and probing fibril formation by dot blotting using the fibril-specific OC antibody (fig. S3C).

### The selected cyclic heptapeptides inhibit Aβ42 aggregation and toxicity in vivo

**Fig. 5:**
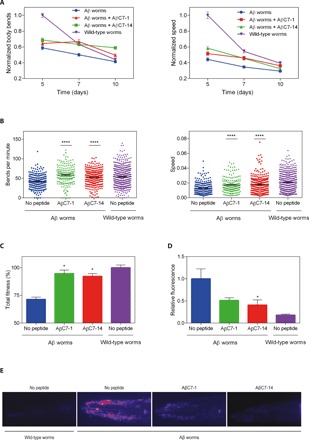
AβC7-1 and AβC7-14 inhibit Aβ42 aggregation in vivo. (**A**) Normalized motility (left) and normalized speed of movement (right) of Aβ42 and wild-type worms in the absence and presence of 40 μΜ AβC7-1 and 5 μΜ AβC7-14 during days 5 to 10 of adulthood. (**B**) Motility (left) and speed (right) of individual Aβ and wild-type worms in the absence and presence of AβC7-1 and AβC7-14 at day 7 of adulthood. (**C**) Total fitness (*51*) of the worms as in (B). (**D**) Relative fluorescence of Aβ42 and wild-type worms at day 7 of adulthood showing a 50 to 60% decrease in Aβ42 aggregate formation in the presence of AβC7-1 and AβC7-14. (**E**) Representative images from (D). In (A) to (C), ~200 worms were analyzed on average, while in (D), 25 worms were analyzed in total. In all panels, mean values ± SEM are presented (*n* = number of worms tested in one experiment). Statistical significance is denoted by *P ≤ 0.05 and ****P ≤ 0.0001, for differences to the “No peptide Aβ worms” sample.

To evaluate the effects of AβC7-1 and AβC7-14 in vivo, we tested their impact on Aβ42 aggregation and Aβ42-induced pathogenicity in an established *C. elegans* model of Alzheimer’s disease. We used GMC101, a transgenic strain expressing human Aβ42 in body wall muscle cells under the control of a heat-inducible promoter (*31*). Upon temperature upshift, these nematodes (hereafter referred to as Aβ worms) exhibit muscle-localized Aβ42 aggregation and eventually the emergence of a paralysis phenotype (*31*). Since the in vitro results suggested that the two compounds affect the early stages of Aβ42 aggregation, AβC7-1 and AβC7-14 were administered to the Aβ worms before aggregation was initiated. The fitness of the Aβ worms—defined as the frequency and speed of body bends—was monitored in the absence and presence of AβC7-1 and AβC7-14 and compared to wild-type nematodes, which do not express Aβ42. Both peptides increased the motility and speed of the Aβ worms throughout their lifetime (Fig. 5, A and B). Both peptides were able to restore the total fitness of the Aβ worms to approximately the levels of the wild-type animals (Fig. 5C). Furthermore, Aβ worms treated with either one of the selected cyclic peptides produced 50 to 60% fewer Aβ42 aggregates, as determined by imaging of the worms using the amyloid-specific dye 2-{[5ʹ-(4-hydroxyphenyl)(2,2ʹ-bithiophen)-5-yl]-methylene}-propanedinitrile (NIAD-4) (Fig. 5, D and E).

To exclude the possibility of promoter- or strain-specific effects, we also treated the transgenic *C. elegans* strain CL4176 with AβC7-1 and AβC7-14, which expresses human Aβ42 in its body wall muscle cells under a different promoter (*32*). Consistent with our previous observations, the administration of both cyclic peptides resulted in a significant delay in the emergence of its characteristic paralysis phenotype (fig. S4). These results demonstrate the protective effect of the two cyclic peptides in the context of an animal, as shown by decrease of Aβ42 deposits, increased locomotion, delay of paralysis, and recovery of total fitness.

### Structure-activity relationships of AβC7-1 and AβC7-14

**Fig. 6:**
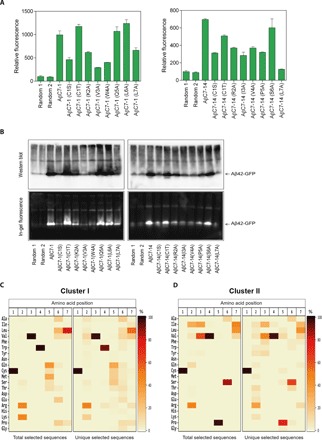
Structure-activity analysis of the selected heptapeptides ΑβC7-1 and AβC7-14. (**A**) Relative fluorescence of *E. coli* Tuner (DE3) cells overexpressing Aβ42-GFP and AβC7-1 (left) or AβC7-14 (right) or the indicated variants thereof as measured by flow cytometry. The fluorescence of the bacterial population coproducing the random cyclic peptide was arbitrarily set to 100. Experiments were carried out in triplicate (*n* = 1 experiment), and the reported values correspond to the mean value ± SEM. (**B**) Western blotting using the anti-Aβ antibody 6E10 (top) and in-gel fluorescence (bottom) analyses following native PAGE of total lysates of *E. coli* Tuner (DE3) cells coexpressing Aβ42-GFP and AβC7-1 (left) or AβC7-14 (right) along with the indicated variants thereof. (**C**) Heatmap representation of the amino acid distribution at each position of the peptide sequences corresponding to cluster I (Fig. 3B), as demonstrated by the deep sequencing analysis results. The total (left) or the unique (right) heptapeptide sequences were included in the analysis. (**D**) As in (C) for cluster II.

To identify the functionally important residues within the selected peptides, we performed nucleophile substitutions at position 1 and Ala-scanning mutagenesis at positions 2 to 7 for both AβC7-1 and AβC7-14. Then, we compared the effects of these amino acid substitutions on the levels of bacterially expressed Aβ42-GFP fluorescence and aggregation with those of the selected sequences (positive control) and of random cyclic peptide sequences (negative control). For both AβC7-1 and AβC7-14, the substitution of Cys at position 1 with Ser resulted in ~50% reduction in fluorescence, while the substitution with Thr resulted in levels of Aβ42-GFP fluorescence and aggregation similar to those corresponding to the selected sequence (Fig. 6, A and B). The latter observation is somewhat unexpected, considering the dominant appearance of Cys^1^ sequences among the selected cyclic heptapeptide pool (Fig. 3A, left), but it may be related to our previous results, where Thr played a crucial role in the identified cyclic peptides against Aβ42 aggregation (*18*). Since the isolation of the bioactive sequences in our system requires repeated rounds of bacterial culturing, protein overexpression and FACS, the scarcity of Thr^1^-containing sequences in the isolated cyclic heptapeptide pool may be occurring because of a toxicity effect of these sequences on bacterial growth, which can result in de-enrichment of the clones that produce them, despite their efficiency in preventing protein aggregation.

Furthermore, for both peptides, Ala-scanning mutagenesis at the majority of the positions 2 to 7 resulted in markedly Aβ42-GFP fluorescence decrease and concomitant increase in aggregation (Fig. 6, A and B). Specifically, for AβC7-1, substitutions at positions 2, 3, 4, and 7 resulted in a ~30 to 70% decrease in Aβ42-GFP fluorescence, while for AβC7-14, substitutions at all positions except Ser^6^ resulted in a ~45 to 80% decrease (Fig. 6, A and B). These observations indicate that a number of residues in both selected cyclic heptapeptides are important for optimal aggregation inhibition activity. When we performed sequence analysis of all the selected sequences belonging to either cluster I or cluster II, we found that the peptides appearing most frequently in each cluster have strong preferences for specific amino acids at each position. More specifically, for cluster I, Arg and Lys at position 2 appeared in >90% of the selected peptides, while Val at position 3, Trp at position 4, Gln, Cys, Ser, Met, and Thr at position 5, and Ile, Val, and Leu at position 7 appeared in >99% of the selected clones (Fig. 6C and table S4). Similarly, for cluster II, the frequency of appearance of Arg, Ile, Val, and Gln at position 2 was ~93%, whereas for Ile and Val at position 3, Val at position 4, Pro at position 5, Ser and Ala at position 6, and Ile, Leu, and Val at position 7, the frequency of appearance was >97% (Fig. 6D and table S4). Together, our results indicate that the most bioactive motifs against Αβ misfolding and aggregation in the investigated macrocycle library are cyclo-(C,T) (R,K)VW (Π,A,M)X (Ψ,P) and cyclo-(C,T)Δ (I,V)VP (S,A)Ψ for clusters I and II, respectively, where X is any one of the 20 natural amino acids; Π is any one of the polar amino acids Q, C, S, and T; Δ is R, I, V, or Q; and Ψ is any one of the aliphatic amino acids L, V, and I.

## DISCUSSION

We have reported how a previously developed bacterial platform can be expanded to enable the simultaneous production and functional screening of molecular libraries with greatly increased diversities for the discovery of inhibitors of disease-associated protein aggregation. We have generated a complete combinatorial library of nearly 200 million head-to-tail cyclic heptapeptides in the cytoplasm of *E. coli* cells and have rapidly screened them to discover inhibitors of the pathogenic misfolding and aggregation of Αβ42. We thus found head-to-tail cyclic heptapeptides that efficiently reduce Aβ42 aggregation and toxicity both in vitro and in vivo. Our highly effective screening methodology, coupled with high-throughput sequencing analysis of the isolated hits, enabled the identification of >400 cyclic heptapeptide putative inhibitors of Aβ42 aggregation. In addition, these results provide further support to our previous observations that low–molecular weight peptide macrocycles are a very rich source of chemical rescuers of protein misfolding (*18*) and that they may constitute a promising class of potential therapeutics (*33*).

Our unbiased selection process yielded distinct groups of bioactive macrocyclic peptides with different sequence characteristics. For the two most dominant clusters, we used a combination of site-directed mutagenesis and deep sequencing analyses to rapidly define the sequence motifs providing optimal bioactivity. These were found to be cyclo-(C,T) (R,K)VW (Π,A,M)X (Ψ,P) for cluster I and cyclo-(C,T)Δ (I,V)VP (S,A)Ψ for cluster II, where X is any one of the 20 natural amino acids; Π is any one of the polar amino acids Q, C, S, or T; Δ is R, I, V, or Q; and Ψ is any one of the aliphatic amino acids L, V, or I. Our in vitro validation indicated that these macrocyclic peptides likely exert protective effects by interfering with microscopic reaction steps underlying the aggregation of Aβ, which affect the generation of oligomers over time. In the context of an in vivo system, as observed in *C. elegans*, where aggregation proceeds on a far longer time scale, this delay in aggregation is much more pronounced and can be considered as effective as an overall arrest of the entire process (*34*).

To our knowledge, the present work describes the largest screen of small molecule–like molecular entities with the ability to perform direct functional screening beyond simple detection of binding to the target protein described to date. Compared to other reported functional compound screens for misfolding rescuing or other bioactivities in vitro or in vivo (*8*, *18*, *25*), we have demonstrated that the system that we described has the ability to generate and evaluate molecular libraries with 20 to 1000 higher diversity than what can be currently achieved. Furthermore, as the diversity of the generated peptide macrocycle libraries are limited only by the theoretical diversity of the library design and the transformation efficiency of *E. coli* cells, our system can allow the evaluation of libraries with tens or even hundreds of billions of members. Notably, *E. coli* can support the biosynthesis of not only head-to-tail cyclic peptides, as investigated here, but also side chain–to–tail cyclic peptides (*35*), bicyclic peptides (*36*), lasso peptides (*37*), θ-defensins (*38*), cyclotides (*39*), and other macrocyclic structures (*40*) that include both natural and noncanonical amino acids (*41*). Contrary to other approaches that allow the investigation of even wider areas of molecular space, such as mRNA display (*10*) and DNA-encoded libraries (*7*, *42*), our technology goes beyond simple detection of binding to the target protein and, instead, selects directly for compounds rescuing aggregation. This is an important advantage, since compound resynthesis and testing for the desired bioactivity following affinity-based selections of DNA- and genetically encoded libraries is time consuming, expensive, and results in a high discovery rate of binders that do not exhibit the desired biological activity (*42*).

It is noteworthy that the sequences of the Αβ42-targeting cyclic heptapeptide discovered here diverge completely from those isolated from our previous screen that included combinatorial libraries of shorter cyclopeptides (*18*). This result suggests that, apart from the specific amino acid residues in the primary sequence of the macrocyclic peptide interacting directly with the target protein and are necessary for bioactivity (*18*), there is probably a conformational component that is also important for molecular recognition between these macrocycles and their targets and that larger cyclopeptide scaffolds are not mere extensions of shorter bioactive sequences. Furthermore, the selected macrocycles bear no resemblance with the sequence of Αβ42, and thus, their discovery would have been very challenging using rational or computationally guided design as, for example, in the case of classical β sheet breaker peptides (*43*) and other designed peptide-based inhibitors of Aβ aggregation (*44*, *45*). Last, also note that the selected cyclopeptides have drug-like molecular characteristics, when compared to those of existing macrocyclic drugs and, in some aspects, to those of conventional drugs as well (table S5).

Our biotechnological approach for producing and evaluating molecular libraries with expanded diversities is not restricted to Αβ42 but is highly versatile and can be applied broadly for targeting a variety of misfolding-prone proteins of both globular and intrinsically disordered nature, as we have shown previously (*18*). We are currently using this system to screen molecular libraries with expanded diversities, such as the ones described here, and have identified candidate macrocyclic rescuers of the misfolding and aggregation of variants of human Cu/Zn superoxide dismutase and p53, as well as of huntingtin, whose misfolding and aggregation are associated with amyotrophic lateral sclerosis, cancer, and Huntington’s disease, respectively (*1*).

The biosynthetic production of the lower-weight molecular libraries under investigation and their simultaneous screening for bioactivity in a simple bacterium like *E. coli* offer great simplicity and speed and reduces the overall cost of the discovery process markedly (*7*, *12*). Once the peptide macrocycle library has been constructed, one can identify the entire repertoire of aggregation inhibitors for a target protein and, at the same time, acquire an initial understanding of structure-activity relationships for the acquired hits in less than a month. The simplicity, speed, and wide applicability of this approach could permit academic and industrial laboratories to simultaneously perform parallel screenings against multiple targets and to prioritize further compound development according to the number and nature of the hits uncovered by the screen. Overall, our approach represents a highly adaptable strategy for investigating molecular libraries with expanded diversities, which enables the discovery of New molecular entities that effectively target peptides and proteins associated with protein misfolding diseases.

## MATERIALS AND METHODS

### Construction of the combinatorial cyclic heptapeptide library

The vector sublibraries pSICLOPPS-CysX_1_X_2_X_3_X_4_X_5_X_6_, pSICLOPPS-SerX_1_X_2_X_3_X_4_X_5_X_6_, and pSICLOPPS-ThrX_1_X_2_X_3_X_4_X_5_X_6_ (table S6) were generated as described previously (*18*). Briefly, the degenerate forward primers GS078, GS079, and GS080 were used together with the reverse primer GS035 and pSICLOPPS as a template (table S6). Cys, Ser, and Thr were encoded in these primers by the codons TGC, AGC, and ACC, respectively, while the randomized amino acids (X) were encoded using random NNS codons, where N = A, T, G, or C and S = G or C. A second PCR reaction was performed in each case to eliminate mismatches using the aforementioned amplified DNA fragments as templates and the forward primers GS069, GS070, and GS071 for each of the peptide sublibraries starting with Cys, Ser, or Thr, respectively, together with the reverse primer GS035. The resulting PCR products were then digested with Bgl I and Hind III for 5 hours and inserted into a similarly digested and dephosphorylated pSICLOPPSKanR vector (*18*). The ligation reactions were optimized at a 12:1 insert:vector ratio and performed at 16°C for 4 hours. Approximately 10 μg of the pSICLOPPSKanR vector was used for each sublibrary. The ligated DNA was then purified using spin columns, transformed into electrocompetent MC1061 cells, plated onto LB agar plates containing chloramphenicol (25 μg/ml), and incubated at 37°C for 14 to 16 hours. This process resulted in approximately 1.2 billion independent transformants, as judged by plating experiments after serial dilutions.

### Cyclic heptapeptide library screening

Electrocompetent *E. coli* Tuner (DE3) cells (Novagen, USA) carrying the expression vector pETAβ42-GFP (*24*) were cotransformed with the combined pSICLOPPS-NuX_1_X_2_X_3_X_4_X_5_X_6_ vector library. Approximately 10^9^ transformants carrying both vectors were harvested, pooled together, and diluted to an OD_600_ of 0.1 in LB liquid medium containing 0.005% l(+)-arabinose to induce cyclic peptide production. Cultures were incubated at 37°C with shaking until an OD_600_ of 0.4 to 0.5, at which point 0.1 mM IPTG was added to the medium to induce overexpression of the Aβ42-GFP reporter. Fluorescence of 50,000 cells was recorder after 2 hours of induction at 37°C using a BD FACSAria II system (BD Biosciences, USA) with a 488-nm solid-state laser for the excitation of GFP and a 530/30 band-pass filter for detection. Then, ~3 × 10^9^ cells were gated on a side-scatter (SSC-H) versus forward-scatter (FSC-H) plot to eliminate noncellular events and were subjected to FACS for the isolation of the bacterial population exhibiting the top ~2% fluorescence. The isolated cells were regrown and screened for six additional rounds in an identical manner, at which point DNA was isolated from the enriched pool using a Qiagen Plasmid Mini Kit.

### High-throughput sequencing analysis

High-throughput sequencing analysis was performed at the Genomics Core Facility of the Biomedical Sciences Research Center “Alexander Fleming” (Athens, Greece) using an Ion Torrent high-throughput sequencing platform. Briefly, the combined pSICLOPPS-NuX_1_X_2_X_3_X_4_X_5_X_6_ vector library and the enriched peptide library after the seventh round of sorting were digested with Nco I and BsrG I, and the resulting ~250 base pair (bp) products that contained the variable peptide-encoding region were isolated and analyzed. Ion proton reads were aligned to a reference sequence using Bowtie2 (v2.2.8). The alignment information stored in the CIGAR string of the resulting Sequence Alignment Map file was parsed and mapped to matching and mismatching sequences using the tool Biostar59647 of the JVarkit utilities. From the resulting XML file, a custom awk script extracted the mismatching insert sequences, which were then clustered using the CD-HIT tool (v4.6.1) (*46*), together with their read counts. From the obtained data, only the 21-bp-long peptide-encoding sequences with NNS codons were subjected to further analysis. For the enriched peptide library, all sequences including stop codons were also discarded from subsequent analysis.

### Peptide sequence similarity analysis and clustering

Sequence similarity analysis was performed using the Immune Epitope Database clustering tool (http://tools.iedb.org/cluster2/) and the fully interconnected clusters (cliques) method (*47*). This approach allows all peptides in a clique to share a minimal level of identity, while at the same time, one peptide can be part of multiple cliques (*47*). As sequence similarity analysis was performed using linear sequences, the circular permutants of each cyclic heptapeptide appearing at least 20 times within the sorted population were identified and taken into consideration, tallying up to 2912 linear representations for the 416 cyclic heptapeptides. From this analysis, 5087 cliques sharing at least 70% sequence identity were identified, and after reintegration of the different circular permutants to their original cyclic peptide sequence, 617 unique cliques remained. From the 416 distinct cyclic heptapeptides, 323 were covered in the cliques forming a total of 1467 unique pairs with more than 70% sequence identity. The remaining 93 cyclic peptides did not share a minimal level of 70% identity with any other of the peptides. The results were then presented in an undirected network graph using the Gephi graph visualization software (*48*), and cluster identification was performed using the Girvan-Newman Algorithm (*49*).

### Aβ42 aggregation kinetic experiments

Kinetic experiments were performed as described previously (*30*). Briefly, appropriate amounts of the synthetic cyclic peptides were added to 2 μM of monomeric Aβ42 to obtain the desired cyclic peptide:Αβ42 molar ratios, and samples were supplemented with 20 μM ThT, 1% (v/v) acetonitrile, and 0.025% or 0.1% (v/v) Tween 20 for ΑβC7-1 and AβC7-14, respectively. Under these conditions, both ΑβC7-1 and AβC7-14 remained stable in a monomeric state for the duration of the in vitro experiments, as judged by dynamic light scattering analyses. All samples were prepared in low-binding Eppendorf tubes on ice using careful pipetting to avoid introduction of air bubbles, and each sample was pipetted into three wells of a 96-well half-area, low-binding, clear-bottom, polyethylene glycol-coated plate (Corning 3881), at 80 μl per well. The 96-well plate was then placed at 37°C under quiescent conditions on a plate reader (Fluostar Omega, Fluostar Optima, or Fluostar Galaxy; BMG Labtech), and after excitation at 440 nm, ThT fluorescence was measured at 480 nm, through the bottom of the plate.

### *C. elegans* motility

#### *Strains*

The following strains were used for this experiment: (i) GMC101, herein referred to as Aβ worms; genotype dvIs100 [unc-54p::A-beta-1-42::unc-54 3′-UTR + mtl-2p::GFP]; mtl-2p::GFP constitutively expresses the GFP in intestinal cells; unc-54p::A-beta-1-42 expresses Aβ42 in body wall muscle cells, resulting in Aβ42 aggregation and worm paralysis after temperature upshift from 20° to 25°C (*31*). (ii) N2, wild-type *C. elegans* var Bristol, herein referred to as wild-type worms (*50*).

#### *Propagation procedures*

*C. elegans* worms were propagated using standard conditions and as described previously (*30*, *50*). Briefly, the worms were treated with hypochlorite bleach, and eggs were hatched overnight in M9 buffer [KH_2_PO_4_ (3 g/liter), Na_2_HPO_4_ (6 g/liter), NaCl (5 g/liter), and 1 mM MgSO_4_] and then distributed on nematode growth medium (NGM) [1 mM CaCl_2_, 1 mM MgSO_4_, cholesterol (5 mg/ml), 250 mM KH_2_PO_4_ (pH 6), agar (17 g/liter), NaCl (3 g/liter), and casein (7.5 g/liter)] plates seeded with the *E. coli* OP50 cells and incubated at 20°C. Upon reaching the L4 stage, ~700 worms were placed on NGM plates containing the desired concentration of the cyclic peptides in 1% (v/v) acetonitrile. Synthetic cyclic peptides were provided to the nematodes as is, without any additional steps to enhance their permeability. At that point, 75 μM 5-fluoro-2′deoxyuridine was also added to the plates to inhibit growth of offspring. The plates were then transferred to 24°C to promote Aβ42 expression and aggregation.

#### *Motility assay*

On days 5 to 10 of adulthood, worms were collected using M9 buffer and distributed on unseeded 9-cm NGM plates. The worms’ movements were recorded at 30 frames/s for 1 min using a homemade microscopic setup, and the body bends were quantified using a custom-tracking algorithm as described previously (*30*, *51*). In total, ~2300 worms were analyzed per peptide with an average of ~200 worms per experiment. Total fitness refers to the sum of the mobility and speed of the worms.

#### *Aggregate quantification*

Staining and microscopy were performed as described previously (*30*). Briefly, live animals were stained by incubating with 1 μM NIAD-4 [0.1% (v/v) dimethyl sulfoxide in M9 buffer] for 6 hours at room temperature and then transferred on NGM plates to allow destaining for about 16 hours. Stained worms were then anesthetized by adding 40 mM NaN_3_ and mounted on 2% agarose pads on glass microscope slides. Images were captured using a Zeiss Axio Observer D1 fluorescence microscope (Carl Zeiss Microscopy GmbH) with a 20× objective and a 49004 ET-CY3/TRITC filter (Chroma Technology Corp.), and fluorescence intensity was calculated using the ImageJ software (National Institutes of Health). Only the head region of the worms was examined because of the high background signal in the intestine.

### Statistical analyses

Statistical analyses were performed using Prism (GraphPad Software Inc., La Jolla, CA, USA), and mean values were compared using unpaired two-tailed *t* tests. For animal experiments, group sizes were chosen on the basis of prior experience and literature precedence so that sufficient numbers remained at the endpoints of the experiment. No samples, worms, or data points were excluded from the reported analyses.

## Supplementary Material

http://advances.sciencemag.org/cgi/content/full/5/10/eaax5108/DC1

Download PDF

Bacterial production and direct functional screening of expanded molecular libraries for discovering inhibitors of protein aggregation

## SUPPLEMENTARY MATERIALS

Supplementary material for this article is available at http://advances.sciencemag.org/cgi/content/full/5/10/eaax5108/DC1 Section S1. Supplementary Materials and Methods Fig. S1. Identification of potential Aβ42 aggregation inhibitors using a bacterial genetic screen. Fig. S2. Identification of different cyclic peptide clusters appearing in the sorted population. Fig. S3. ΑβC7-1 and AβC7-14 inhibit the aggregation of Aβ42 in vitro. Fig. S4. ΑβC7-1 and AβC7-14 inhibit the aggregation of Aβ42 in vivo. Table S1. Deep sequencing analysis of the peptide-encoding regions of ~3.4 million clones from the constructed pSICLOPPS-NuX_1_X_2_X_3_X_4_X_5_X_6_ library. Table S2. Enrichment (blue) and depletion (red) of the 20 amino acids in each position of the heptapeptide sequences. Table S3. Distribution of the heptapeptide sequences in the different clusters identified. Table S4. Sequences and frequency of appearance of cluster I and cluster II heptapeptide sequences as determined by high-throughput sequencing of the enriched library after the seventh round of sorting. Table S5. Molecular properties of the selected cyclic heptapeptides AβC7-1 and AβC7-14 compared to those of conventional drugs, oral macrocyclic (MC) drugs, and nonoral MC drugs. Table S6. Plasmids and PCR primers used in this study. References (*52*, *53*)

## Appendix

View/request a protocol for this paper from *Bio-protocol*.
