# Supplementary material for: Bacterial production and direct functional screening of expanded molecular libraries for discovering inhibitors of protein aggregation
Source: Sci Adv. 2019 Oct 16;5(10):eaax5108. doi: 10.1126/sciadv.aax5108 (PMC6795521; doi:10.1126/sciadv.aax5108)
Supplement: http://advances.sciencemag.org/cgi/content/full/5/10/eaax5108/DC1 [file supp_5_10_eaax5108__index.html]

Science Advances | Science AdvancesAAASSearchScience AdvancesMenu

## Supplementary Materials

**This PDF file includes:**

- Section S1. Supplementary Materials and Methods
- Fig. S1. Identification of potential Aβ42 aggregation inhibitors using a bacterial genetic screen.
- Fig. S2. Identification of different cyclic peptide clusters appearing in the sorted population.
- Fig. S3. ΑβC7-1 and AβC7-14 inhibit the aggregation of Aβ42 in vitro.
- Fig. S4. ΑβC7-1 and AβC7-14 inhibit the aggregation of Aβ42 in vivo.
- Table S1. Deep sequencing analysis of the peptide-encoding regions of ~3.4 million clones from the constructed pSICLOPPS-NuX1X2X3X4X5X6 library.
- Table S2. Enrichment (blue) and depletion (red) of the 20 amino acids in each position of the heptapeptide sequences.
- Table S3. Distribution of the heptapeptide sequences in the different clusters identified.
- Table S4. Sequences and frequency of appearance of cluster I and cluster II heptapeptide sequences as determined by high-throughput sequencing of the enriched library after the seventh round of sorting.
- Table S5. Molecular properties of the selected cyclic heptapeptides AβC7-1 and AβC7-14 compared to those of conventional drugs, oral macrocyclic (MC) drugs, and nonoral MC drugs.
- Table S6. Plasmids and PCR primers used in this study.
- References (*52*, *53*)

Download PDF

**Files in this Data Supplement:**

- Adobe PDF - aax5108\_SM.pdf
