# Supplementary material for: Bacterial production and direct functional screening of expanded molecular libraries for discovering inhibitors of protein aggregation
Source: Sci Adv. 2019 Oct 16;5(10):eaax5108. doi: 10.1126/sciadv.aax5108 (PMC6795521; doi:10.1126/sciadv.aax5108)
Supplement: Download PDF [file aax5108_SM.pdf]

Science Advances
AAAS

advances.sciencemag.org/cgi/content/full/5/10/eaax5108/DC1

Supplementary Materials for

Bacterial production and direct functional screening of expanded molecular libraries for discovering inhibitors of protein aggregation

Dafni C. Delivoria, Sean Chia, Johnny Habchi, Michele Perni, Ilias Matis, Nikoletta Papaevgeniou, Martin Reczko, Niki Chondrogianni, Christopher M. Dobson, Michele Vendruscolo, Georgios Skretas*

*Corresponding author. Email: gskretas@eie.gr

Published 16 October 2019, *Sci. Adv.* **5**, eaax5108 (2019)
DOI: 10.1126/sciadv.aax5108

This PDF file includes:

Section S1. Supplementary Materials and Methods
Fig. S1. Identification of potential Aβ42 aggregation inhibitors using a bacterial genetic screen.
Fig. S2. Identification of different cyclic peptide clusters appearing in the sorted population.
Fig. S3. AβC7-1 and AβC7-14 inhibit the aggregation of Aβ42 in vitro.
Fig. S4. AβC7-1 and AβC7-14 inhibit the aggregation of Aβ42 in vivo.
Table S1. Deep sequencing analysis of the peptide-encoding regions of ~3.4 million clones from the constructed pSICLOPPS-Nu$X_1X_2X_3X_4X_5X_6$ library.
Table S2. Enrichment (blue) and depletion (red) of the 20 amino acids in each position of the heptapeptide sequences.
Table S3. Distribution of the heptapeptide sequences in the different clusters identified.
Table S4. Sequences and frequency of appearance of cluster I and cluster II heptapeptide sequences as determined by high-throughput sequencing of the enriched library after the seventh round of sorting.
Table S5. Molecular properties of the selected cyclic heptapeptides AβC7-1 and AβC7-14 compared to those of conventional drugs, oral macrocyclic (MC) drugs, and nonoral MC drugs.
Table S6. Plasmids and PCR primers used in this study.
References (*52*, *53*)

Section S1. Supplementary Materials and Methods

Reagents and chemicals

All enzymes for recombinant DNA used in this study were purchased from New England Biolabs with the exception of alkaline phosphatase FastAP which was obtained for ThermoFisher Scientific. Recombinant plasmids were purified using Macherey-Nagel NucleoSpin Plasmid and Qiagen Plasmid Mini and Midi kits. PCR products and DNA extracted from agarose gels were purified using Macherey-Nagel Nucleospin Gel and PCR Clean-up. Synthetic AβC7-1 and AβC7-14 were purchased from Proteogenix (France). Before each experiment, AβC7-1 was freshly dissolved in 50% acetonitrile/0.1% Tween-20 and AβC7-14 was dissolved in 30% acetonitrile/0.025% Tween-20 to form 10 mM solutions. Under these conditions, both cyclic heptapeptides were stably in a monomeric form for the duration of the *in vitro* experiments, as determined by dynamic light scattering (data not shown).

Plasmid constructions

For the construction of pSICLOPPS(H24L/F26A)-AβC7-1, pSICLOPPS(H24L/F26A)-AβC7-2, pSICLOPPS(H24L/F26A)-AβC7-3, pSICLOPPS(H24L/F26A)-AβC7-7 and pSICLOPPS(H24L/F26A)-AβC7-14, plasmids pSICLOPPS-AβC7-1, pSICLOPPS-AβC7-2 pSICLOPPS-AβC7-3, pSICLOPPS-AβC7-7 and pSICLOPPS-AβC7-14 were digested with BglI and HindIII and the resulting inserts were ligated into a similarly digested pSICLOPPS(H24L/F26A)KanR vector (*18*). For the construction of pSICLOPPS-AβC7-371, pSICLOPPS-AβC7-405 and pSICLOPPS-AβC7-416, forward primers DD175, DD176 and DD178 were used individually, in pair with the reverse primer GS035 and using pSICLOPPS as a template and the resulting PCR products were digested with BglI and HindIII and ligated into a similarly digested pSICLOPPSKanR (*18*). For the construction of

pSICLOPPS-AβC7-1(C1S), pSICLOPPS-AβC7-1(C1T), pSICLOPPS-AβC7-1(K2A), pSICLOPPS-AβC7-1(V3A), pSICLOPPS-AβC7-1(W4A), pSICLOPPS-AβC7-1(Q5A), pSICLOPPS-AβC7-1(L6A), pSICLOPPS-AβC7-1(L7A), pSICLOPPS-AβC7-14(C1S), pSICLOPPS-AβC7-14(C1T), pSICLOPPS-AβC7-14(R2A), pSICLOPPS-AβC7-14(I3A), pSICLOPPS-AβC7-14(V4A), pSICLOPPS-AβC7-14(P5A), pSICLOPPS-AβC7-14(S6A) and pSICLOPPS-AβC7-14(L7A), forward primers DD129, DD130, DD131, DD132, DD133, DD134, DD135, DD136, DD137, DD138, DD139, DD140, DD141, DD142, DD143 and DD144 were used individually, in pair with the reverse primer GS035 and using pSICLOPPS-AβC7-1 or pSICLOPPS-AβC7-14 as a template, accordingly. The resulting PCR products were digested with BglI and HindIII and ligated into a similarly digested pSICLOPPSKanR (*18*). For the construction of pET-Aβ42, the Aβ42 gene was amplified using primers DD004 and IM022 as well as pAβ42-GFP (*24*) as a template. The resulting PCR product was digested with NcoI and XhoI and inserted into a similarly digested pET28a (+) (Novagen, USA).

Protein/cyclic peptide production in liquid cultures

Protein and cyclic peptide overexpression were performed essentially as described previously (*18*). Briefly, *E. coli* Tuner (DE3) cells freshly transformed with the appropriate expression vectors were used for all protein production experiments. Single bacterial colonies were used to inoculate liquid LB cultures containing 40 μg/mL chloramphenicol and 50 μg/mL kanamycin. These cultures were used with a 1:100 dilution to inoculate fresh LB media containing 0.02% L (+)-arabinose which were grown at 37 ^{o}C to an OD_{600} of ~0.4 with shaking, at which point protein production was induced by the addition of 0.1 mM IPTG for 2 h.

Bacterial cell fluorescence

Bulk bacterial fluorescence measurements were carried out as described previously (*18*). Briefly, after protein overexpression, cells corresponding to 1 mL culture with OD_{600}=1 were harvested by centrifugation, re-suspended in 100 μL phosphate-buffered saline (PBS), transferred to a 96-well FLUOTRAC 200 plate (Greiner Bio One International, Austria) and after excitation at 488 nm, their fluorescence was measured at 510 nm using a TECAN Safire II-Basic plate reader (Tecan, Austria). For ThS fluorescence measurements, a similar procedure was followed with the exception that cells were re-suspended in 250 μM ThS in PBS, equilibrated for 15 min and then emission spectra were recorded at a range of 460-600 nm. For flow cytometric measurements, after protein overexpression, fluorescence of 50,000 cells resuspended in PBS was recorder at 530/30 nm after GFP excitation at 488nm, using a BD FACSAria II system (BD Biosciences, USA).

In-gel fluorescence and western blot analyses

After protein overexpression, cells corresponding to 1 mL culture with OD_{600}=1 were harvested by centrifugation and re-suspended in 100 μL PBS. Samples were lysed by brief sonication cycles on ice and the resulting lysates (total fraction) were clarified by centrifugation at 13,000 rpm for 25 min (soluble fraction). Samples were analyzed by native or SDS-PAGE, on 10 or 15% gels respectively and without prior boiling (for in-gel fluorescence) or after boiling of the samples for 10 min (for western blotting). In-gel fluorescence was analyzed on a ChemiDoc-It^2 Imaging System equipped with a CCD camera and a GFP filter (UVP, UK), after exposure for about 3 sec. For western blotting, proteins were transferred to polyvinylidene fluoride (PVDF) membranes (Merck, Germany) for 1 h at 12 V on a semi-dry blotter (Thermo Fisher, USA). Membranes were blocked with 5% non-fat dry milk in Tris-buffered saline containing 0.1% Tween-20 (TBST) for 1 h at room temperature. After washing with TBST three times, membranes were incubated with

the appropriate antibody diluted in 0.5% non-fat dry milk in TBST at room temperature for 1 h. The utilized antibodies were a mouse anti-Aβ (6E10) (Covance, USA) at 1:2,000 dilution, a mouse anti-CBD antibody (New England Biolabs, USA) at 1:100,000 dilution and a horseradish peroxidase-conjugated goat anti-mouse secondary antibody (BioRad) at 1:4000 dilution. The proteins were then visualized using the ChemiDoc-It^2 Imaging System (UVP, UK).

Preparation of Aβ42

The recombinant Aβ (M1-42) peptide (MDAEFRHDSGYEVHHQKLVFFAEDVGSNKG AIIGLMVGGVVIA), herein termed Aβ42, was expressed in *E. coli* and purified as described previously (*30*). The lyophilized peptide was then dissolved in 6 M GuHCl, further purified using a Superdex 75 10/300 GL column (GE Healthcare) and eluted in 20 mM sodium phosphate buffer, pH 8, supplemented with 200 μM EDTA and 0.02% NaN_3. The centre of the peak was collected and Aβ42 concentration was determined from the absorbance of the integrated peak area using $\varepsilon_{280} = 1{,}490\ M^{-1}\ cm^{-1}$.

Transmission electron microscopy

TEM experiments were performed as described previously (*52*). Briefly, 5 μL aliquots from each sample were removed after the aggregation reaction had reached the final plateau and placed on a carbon support film on 400-mesh copper grid (EM Resolutions Ltd.). After adsorption, grids were negatively stained with 2% w/v uranyl acetate and images were recorded using a FEI Tecnai G_2 transmission electron microscope (Cambridge Advanced Imaging Centre) and analyzed using the SIS Megaview II Image Capture system (Olympus).

Dot blots

The dot blot assay was performed as described previously (*30*) while samples were prepared as for the kinetic experiments albeit in the absence of ThT. 2 μL aliquots of each sample were removed at different time points and spotted onto a nitrocellulose membrane (0.2 μm; Whatman). The membranes were then dried, blocked with 5% non-fat dry milk in PBST (0.1%) and incubated with an Aβ42 fibril-specific primary antibody (OC, Millipore) and Alexa-Fluor 488-conjugated secondary antibody (Life Technologies) according to the manufacturer's instructions. Fluorescence detection was performed using the Typhoon Trio Imager (GE Healthcare) and fluorescence quantification was performed using the ImageJ software (National Institutes of Health).

***C. elegans* paralysis**

The paralysis assay was performed using standard procedures and as described previously (*18*). Briefly, synchronized L4 larvae CL4176 {smg-1 (cc546) I; dvIs27 [myo-3::Aβ (1-42)-let 3'UTR (pAF29); pRF4 (rol-6 (su1006)] (*32*)} (~150-300 per condition) were transferred to NGM plates, that were seeded with *E. coli* OP50 and contained 10 μM of the cyclic peptides in 1% acetonitrile, and were incubated at 16 °C for 48 h before transgene induction via temperature up-shift to 25 °C. Synchronized offspring were randomly distributed to treatment plates to avoid systematic differences in egg lay batches. Treatment and control plates were handled, scored and assayed in parallel. Scoring of paralyzed animals was initiated 24 h after temperature up-shift. Worms were considered as paralyzed upon failure to move their half end-body upon prodding, while animals that died were excluded from the assay. The log-rank (Mantel–Cox) test was used to evaluate differences between paralysis curves and to determine *P* values for all independent data. *n* in paralysis figures is the number of animals that paralyzed over the total number of animals used (the number of paralyzed animals plus the number of dead and censored animals). Median paralysis values are expressed as mean ± s.e.m.

Supplementary Figures

A

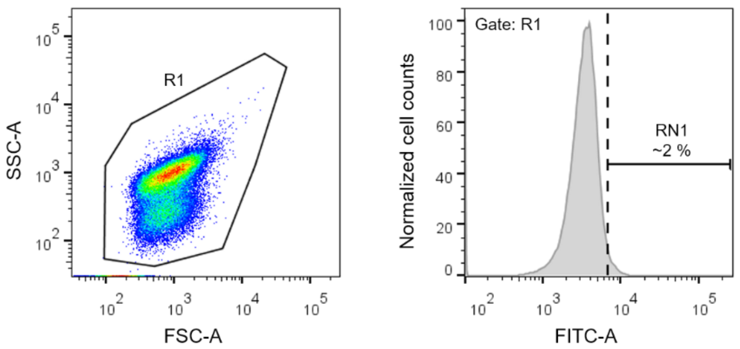

B

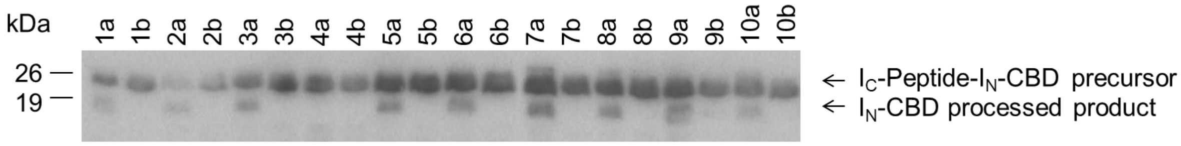

C

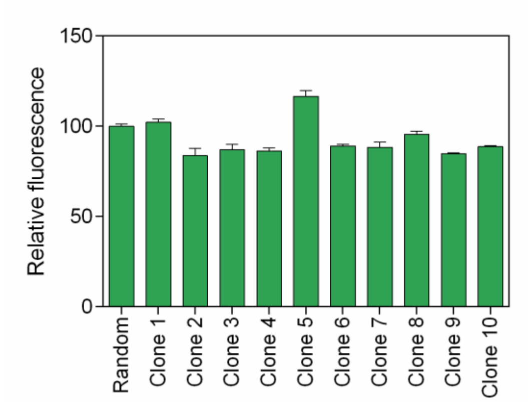

D

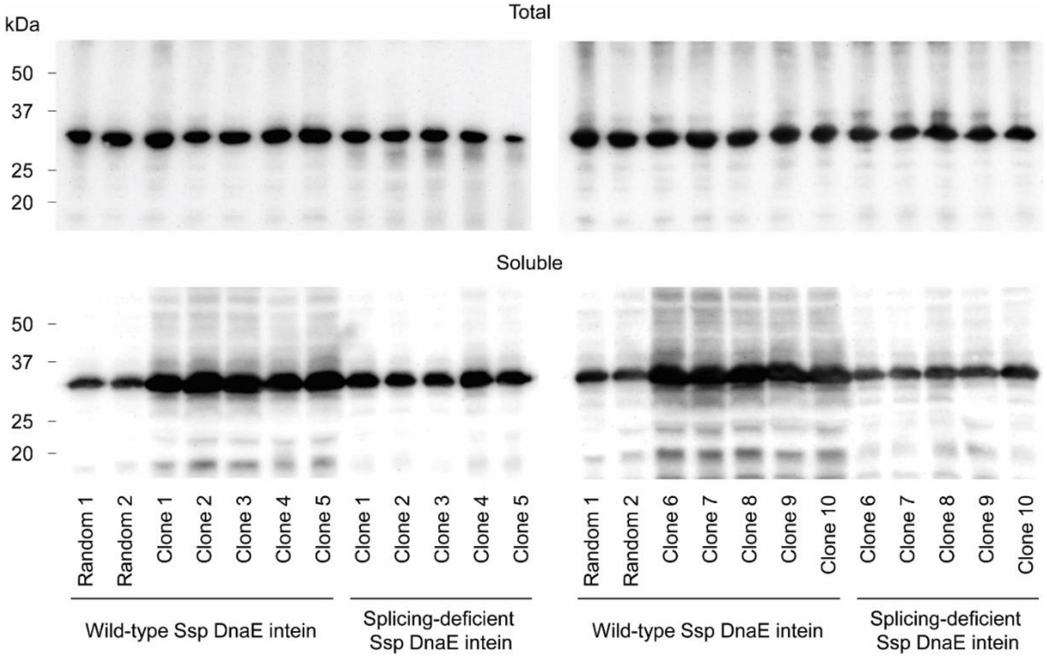

E

Isolated Clone #	DNA sequence of peptide-encoding region	Encoded peptide sequence
1	TGC AGG GTG TGG ACG GAG TTG	CRVWTEL
2	TGC AAG GTG TGG CAG TTG TTG	CKVWQLL
3	TGC AAG GTG TGG CAG TTG TTG	CKVWQLL
4	TGC ATC GTC GTC CCG TCG ATC	CIVVPSI
5	TGC AAG GTC TGG ATG CCG CTC	CKVWMPL
6	TGC AAG GTG TGG CAG TTG TTG	CKVWQLL
7	TGC AAG GTG TGG CAG TTG TTG	CKVWQLL
8	TGC AAG GTG TGG CAG TTG TTG	CKVWQLL
9	TGC CGC ATC GTC CCC AGC TTG	CRIVPSL
10	TGC AAG GTG TGG CAG TTG TTG	CKVWQLL

F

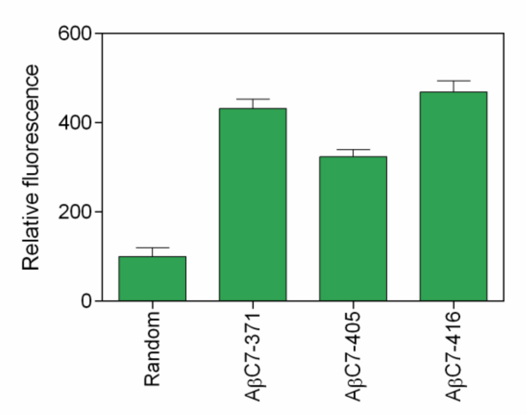

Fig. S1. Identification of potential Aβ42 aggregation inhibitors using a bacterial genetic screen. (**A**) Gating strategy for the isolation of aggregation inhibitors using FACS. (left) Cells were first gated based on their forward and side scatter properties, in order to eliminate non-cellular events. (right) The selected cells were subjected to multiple rounds of FACS sorting, each time isolating the bacterial population exhibiting the top ~2% fluorescence. (**B**) Western blot analysis of the ten selected clones (**Fig. 2C**) using an anti-CBD antibody. The upper band of ~25 kDa corresponds to the I_C-peptide sequence-I_N-CBD precursor, while the lower band of ~20 kDa corresponds to the processed I_N-CBD product, whose appearance is an indication of successful cyclic peptide formation. CBD: chitin-binding domain. For each clone, (a) represents a wild-type intein where cyclic peptide formation is allowed, while (b) represents the H24L/F26A splicing deficient variant where no cyclic peptide is produced. (**C**) Relative fluorescence of *E. coli* Tuner(DE3) cells overexpressing p53(Y220C)-GFP and the ten selected cyclic heptapeptide clones tested in **Fig. 2C**. The fluorescence of the bacterial population producing the random cyclic peptide was arbitrarily set to 100. Experiments were carried out in replica triplicates (*n*=1 independent experiment) and the reported values correspond to the mean value ± s.e.m. **(D)** Western blot analysis of total (top) and soluble (bottom) lysates of *E. coli* Tuner(DE3) cells overexpressing Aβ42-GFP and the ten individual cyclic peptide sequences tested in **Fig. 2C** using either the wild-type split-intein construct or the splicing-deficient variant H24L/F26A

and utilizing the anti-Aβ antibody 6E10 for probing. The predicted molecular mass of the Aβ42-GFP fusion is ~32 kDa. (**E**) Cyclic heptapeptide sequences encoded by the selected bacterial clones exhibiting enhanced Aβ42-GFP fluorescence. **(F)** Relative fluorescence of *E. coli* Tuner(DE3) cells co-expressing Aβ42-GFP and three cyclic heptapeptide sequences appearing in the sorted pool only at low frequencies as shown in **Table S4**. The fluorescence of the cell population producing a random cyclic peptide was arbitrarily set to 100. Experiments were carried out in triplicates (n=1 independent experiments) and the reported values correspond to the mean value ± s.e.m.

A

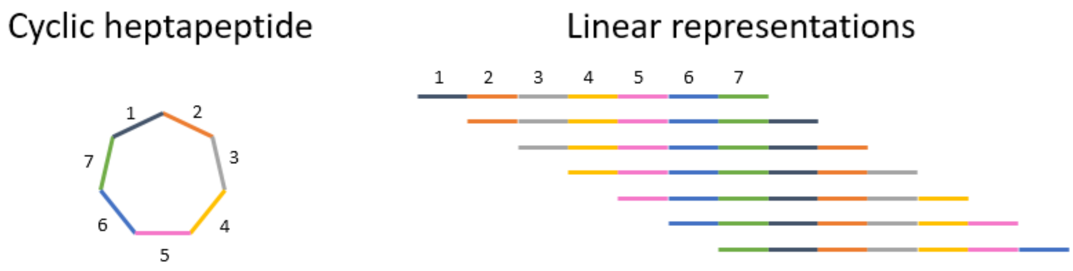

B

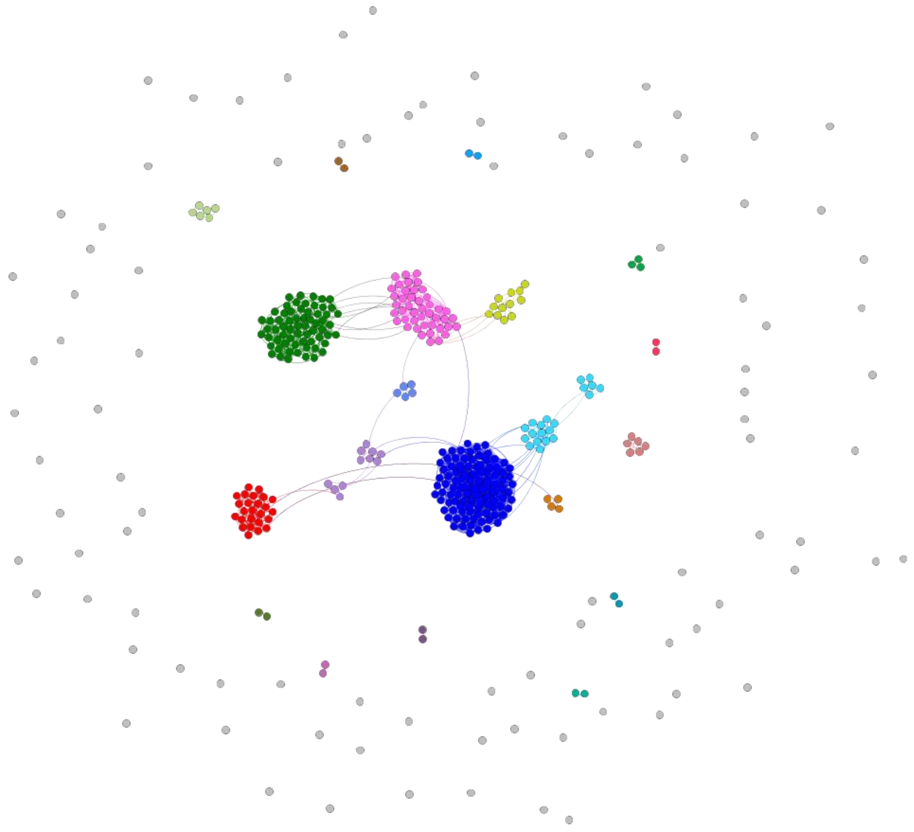

Fig. S2. Identification of different cyclic peptide clusters appearing in the sorted population. (**A**) Schematic of the linear representations (circular permutants) of a cyclic heptapeptide. (**B**) Network visualization of all clusters identified using the Girvan-Newman algorithm of the Gephi software as in **Fig. 3B**. The grey nodes represent cyclic heptapeptides

that do not share at least 70% homology with any other cyclic peptide from the selected pool.

A

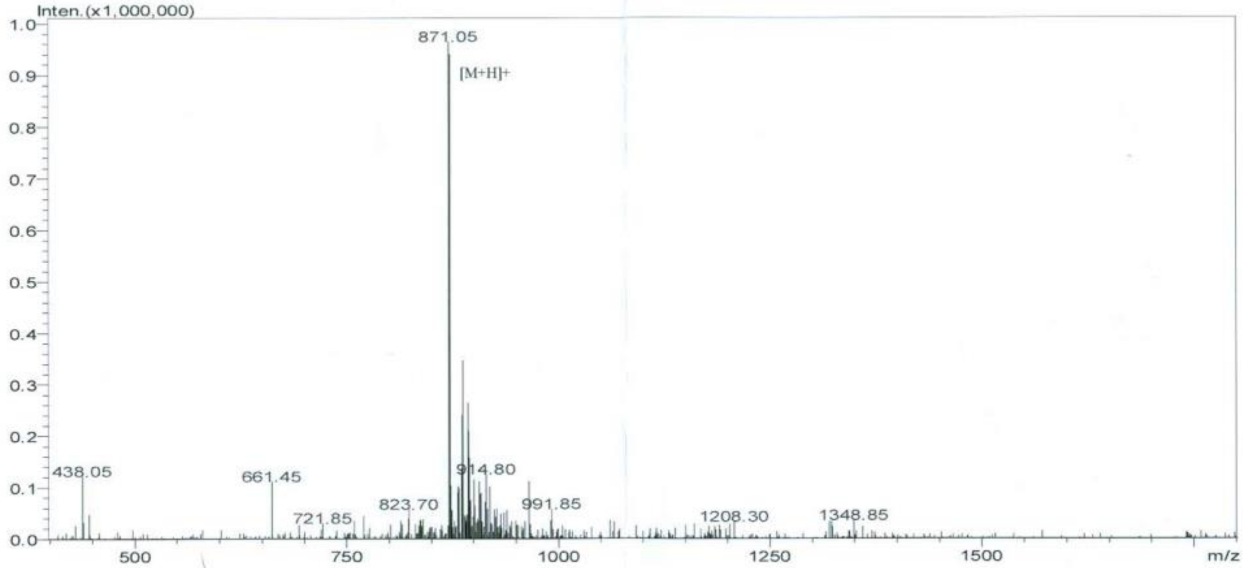

Sample Information		Probe:	ESI	Probe Bias:	+4.5kv
Injection Volume	: 1.00 μ l	Nebulizer Gas Flow:	1.5L/min	Detector:	1.5kv
Sample:	CL-7	CDL:	-20.0v	T. Flow:	0.2ml/min
M.W.:	870.10	CDL Temp.:	250 °C	B. Conc.:	50%H2O/50%ACN
Lot. No.:	P160504-GY502012	Block Temp.:	200 °C		

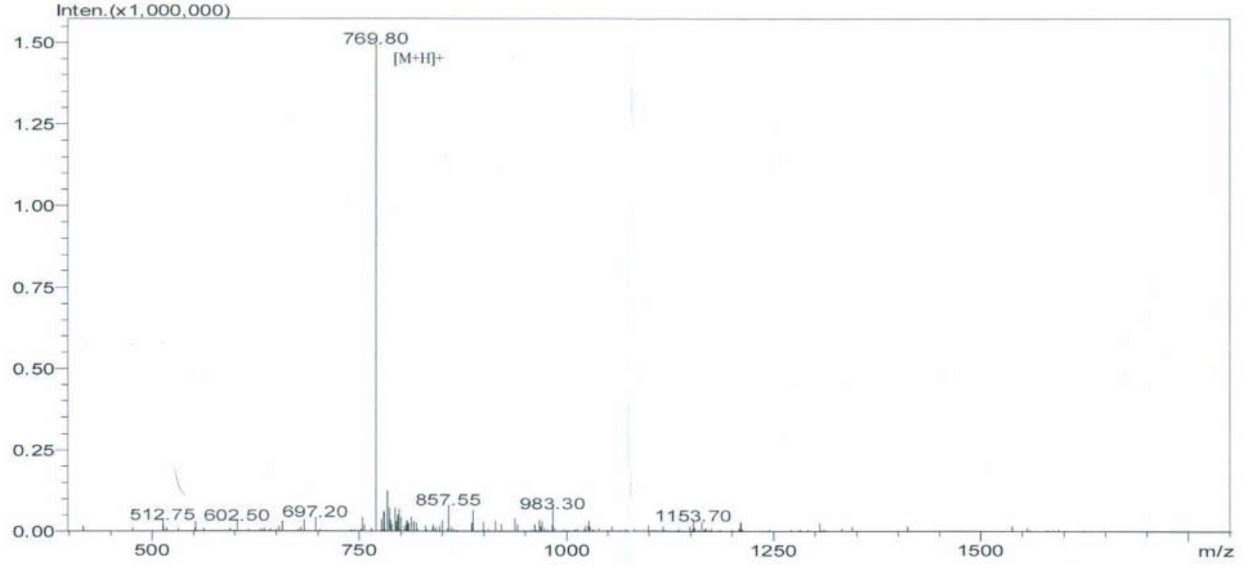

Sample Information		Probe:	ESI	Probe Bias:	+4.5kv
Injection Volume	: 1.00 μ l	Nebulizer Gas Flow:	1.5L/min	Detector:	1.5kv
Sample:	CL-7	CDL:	-20.0v	T. Flow:	0.2ml/min
M.W.:	768.98	CDL Temp.:	250 °C	B. Conc.:	50%H2O/50%ACN
Lot. No.:	P160504-GY502013	Block Temp.:	200 °C		

B

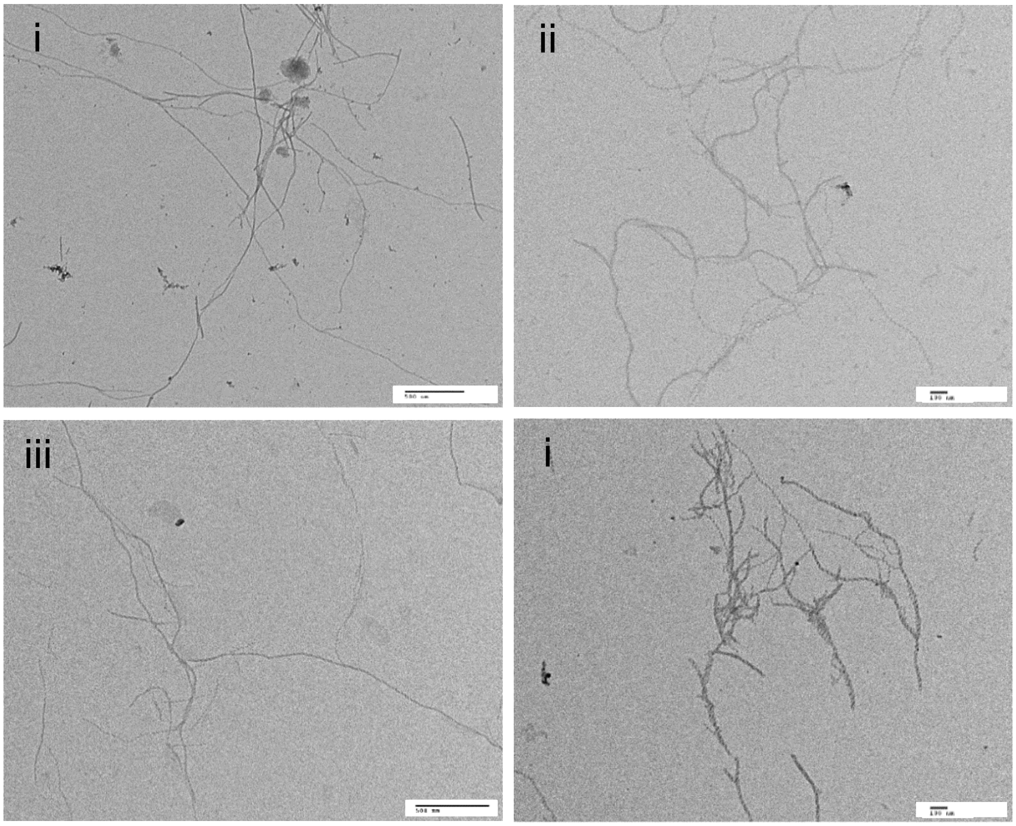

C

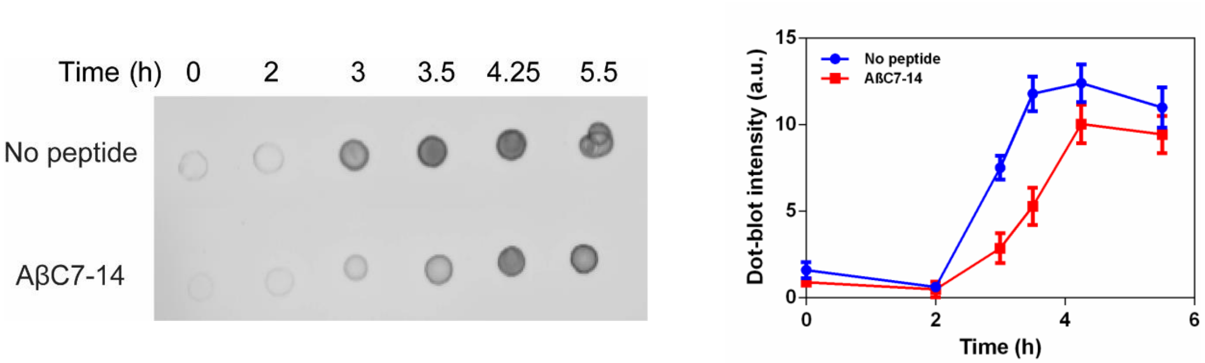

Fig. S3. AβC7-1 and AβC7-14 inhibit the aggregation of Aβ42 in vitro. (**A**) Full scan spectrum (ESI-MS) of the synthesized AβC7-1 (top) and AβC7-14 (bottom) by solid-phase chemical synthesis.(**B**) TEM images of 2 μM Aβ42 fibrils in the absence (left) and presence (right) of either 10 μM AβC7-1 (top) or 4 μM AβC7-14 (bottom). Samples were taken at the reaction end point as measured by thioflavin T. (**C**) Time course of the aggregation of 2 μM Aβ42 in the presence and absence of 0.5 μM AβC7-14 using a dot blot assay and the Aβ42 fibril-specific antibody OC. Mean values ± s.e.m. are presented (n= 1 experiment performed in three replicates).

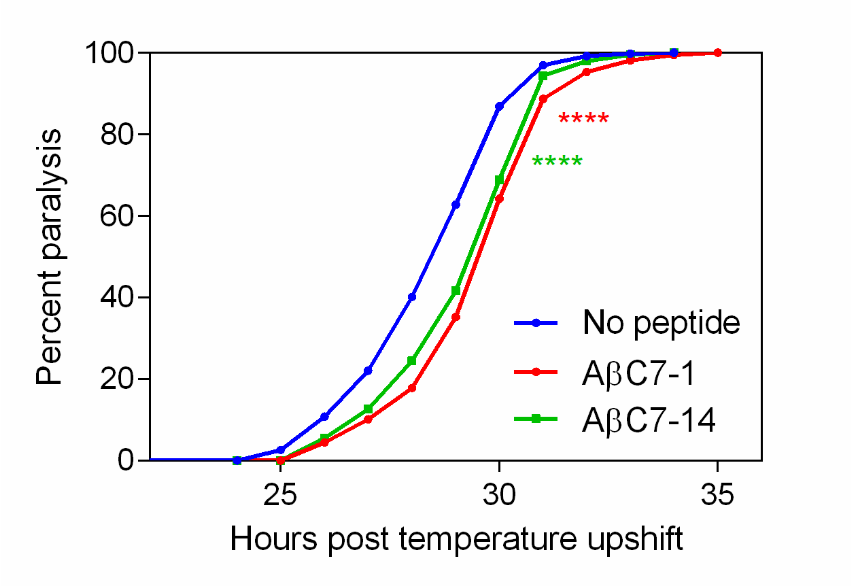

Fig. S4. AβC7-1 and AβC7-14 inhibit the aggregation of Aβ42 in vivo. Paralysis curves of *C. elegans* CL4176 expressing human Aβ42 and treated with 10 μM of synthetic AβC7-1 and AβC7-14. The "No peptide" sample (control) contains the same amount of solvent as the test samples (1% acetonitrile). No peptide: mean=28.4±0.2, n=863/867; AβC7-1: mean=29.5±0.1, n=867/873, P<0.0001; and AβC7-14: mean=29.2±0.1, n=887/893, P<0.0001.

Supplementary Tables

Table S1. Deep sequencing analysis of the peptide-encoding regions of ~3.4 million clones from the constructed pSICLOPPS-Nu$X_1X_2X_3X_4X_5X_6$ library.

	Number of reads	Unique DNA sequences	Unique peptide sequences
cyclo- Cys$X_1X_2X_3X_4X_5X_6$ sub-library	1,305,675	1,023,580 (78%)	978,803 (96%)
cyclo-Ser$X_1X_2X_3X_4X_5X_6$ sub-library	1,318,365	885,393 (67%)	824,134 (93%)
cyclo-Thr$X_1X_2X_3X_4X_5X_6$ sub-library	769,605	652,099 (85%)	636,647 (98%)
**Combined cyclo-Nu$X_1X_2X_3X_4X_5X_6$ library**	**3,393,645**	**2,561,072 (75%)**	**2,439,584 (95%)**

Table S2. Enrichment (blue) and depletion (red) of the 20 amino acids in each position of the heptapeptide sequences. Values represent the log2 fold change of the amino acid distribution of peptides from the selected pool compared to the initial library.

		Position						
		1	2	3	4	5	6	7
Amino Acid	A		-2.4	-5.1	-5	-2.9	0.6	-0.6
	I		-0.5	-0.1	-1.3	0.7	-3.7	1.8
	L		-4.8	-0.3	-3.2	-1.9	1.5	3.3
	V		-1.8	3.2	0.1	-1.6	0.4	0.1
	F		-4.7	-3.9	-4.6	-5	0.2	-0.3
	W		-5.2	-2.9	3.8	-2.1	-1.9	-3.2
	Y		-1.2	-0.3	-4.8	-2.2	0.1	-7.3
	N		-3.5	-2	-3.9	-1.2	-2.1	-7.4
	Q		-0.8	-0.4	-4.9	3.9	0.2	-1.5
	C	1.4	-1.3	-3.4	-5.6	0.9	-2	-4.8
	M		-3	-2.5	-2.5	3.4	-2.1	-2.6
	S	-6.7	-0.9	-1.6	-4.8	0.8	-0.7	-6.7
	T	-7.1	-1.3	-4.2	0.8	1	-0.1	-1.2
	D		-4.7	-4.4	-5.9	-6.6	-2.3	-8.1
	E		-1.8	-3.6	-6.2	-4.7	1.8	-6.9
	R		2.1	-5	-2.5	-6.2	-4.1	-2.7
	H		-4	-5.6	-5.7	-6.2	-0.1	-5.6
	K		4.2	-6.6	-5.6	-0.4	1.5	-3.3
	P		-4.5	-2.9	0	0.4	1.9	0.6
	G		-1.8	-6.2	-5.1	-4.6	-3.4	-6.5

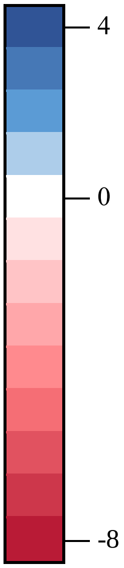

Table S3. Distribution of the heptapeptide sequences in the different clusters identified.

Number	Cluster name	Number of reads	Number of unique peptides	Cluster Reads/Total reads (%)	Peptides/Total peptides (%)
1	I	303,245	107	75.01	25.72
2	II	19,690	25	4.87	6.01
3	III	16,667	64	4.12	15.38
4	IV	6,047	44	1.50	10.58
5	V	5,362	20	1.33	4.81
6	VI	2,837	12	0.70	2.88
7	VII	2,192	6	0.54	1.44
8	VIII	1,987	5	0.49	1.20
9	IX	1,700	2	0.42	0.48
10	X	1,545	6	0.38	1.44
11	XI	1,290	4	0.32	0.96
12	XII	611	2	0.15	0.48
13	XIII	606	11	0.15	2.64
14	XIV	482	2	0.12	0.48
15	XV	258	3	0.06	0.72
16	XVI	235	2	0.06	0.48
17	XVII	95	2	0.02	0.48
18	XVIII	86	2	0.02	0.48
19	XIX	54	2	0.01	0.48
20	XX	42	2	0.01	0.48
21	Singletons	39,249	93	9.71	22.36
**Sum**		**404,280**	**416**	**100**	**100**

Table S4. Sequences and frequency of appearance of cluster I and cluster II heptapeptide sequences as determined by high-throughput sequencing of the enriched library after the seventh round of sorting.

Cluster I											
Number	Peptide name	Amino acid sequence							Number of reads	Reads/ Total Cluster I reads (%)	Reads/Total heptapeptide reads (%)
1	AβC7-1	C	K	V	W	Q	L	L	64118	21.144	15.86
2	AβC7-2	C	K	V	W	M	P	L	42464	14.003	10.504
3	AβC7-3	C	R	V	W	T	E	L	28675	9.456	7.093
4	AβC7-5	C	R	V	W	M	V	P	20170	6.651	4.989
5	AβC7-6	C	R	V	W	C	A	L	18397	6.067	4.551
6	AβC7-7	C	R	V	W	Q	T	V	14267	4.705	3.529
7	AβC7-8	C	S	V	W	M	E	L	12638	4.168	3.126
8	AβC7-9	C	R	V	W	Q	A	L	11718	3.864	2.898
9	AβC7-10	C	K	V	W	Q	V	L	9249	3.05	2.288
10	AβC7-11	C	R	V	W	S	L	L	9124	3.009	2.257
11	AβC7-12	C	K	V	W	M	A	L	7513	2.478	1.858
12	AβC7-13	C	K	V	W	S	Q	L	7166	2.363	1.773
13	AβC7-15	C	R	V	W	Q	L	L	6030	1.988	1.492
14	AβC7-19	C	R	V	W	C	E	L	5223	1.722	1.292
15	AβC7-20	C	K	V	W	M	E	V	5043	1.663	1.247
16	AβC7-22	C	R	V	W	S	P	L	4843	1.597	1.198
17	AβC7-23	C	R	V	W	M	G	L	4212	1.389	1.042
18	AβC7-24	C	T	V	W	M	A	I	3654	1.205	0.904
19	AβC7-26	C	V	V	W	Q	P	L	2611	0.861	0.646
20	AβC7-27	C	R	V	W	Q	V	V	2595	0.856	0.642
21	AβC7-29	C	R	V	W	S	A	L	2327	0.767	0.576
22	AβC7-30	C	A	V	W	Q	A	L	2280	0.752	0.564
23	AβC7-32	C	R	V	W	C	A	V	1971	0.65	0.488
24	AβC7-40	C	K	V	W	C	V	M	1059	0.349	0.262
25	AβC7-41	C	R	V	W	Q	C	V	1028	0.339	0.254
26	AβC7-43	C	A	V	W	M	Q	L	953	0.314	0.236
27	AβC7-49	C	R	V	W	M	M	L	815	0.269	0.202
28	AβC7-50	C	R	V	W	S	V	V	803	0.265	0.199
29	AβC7-51	C	R	V	W	Q	E	V	796	0.262	0.197
30	AβC7-52	C	V	V	W	Q	Q	I	790	0.261	0.195
31	AβC7-58	C	Q	V	W	M	D	L	608	0.2	0.15
32	AβC7-60	C	R	V	W	Q	D	P	575	0.19	0.142
33	AβC7-62	C	R	V	W	M	L	L	486	0.16	0.12
34	AβC7-63	C	V	V	W	Q	L	L	473	0.156	0.117
35	AβC7-67	C	R	V	W	Y	G	I	435	0.143	0.108

Cluster I											
Number	Peptide name	Amino acid sequence							Number of reads	Reads/ Total Cluster I reads (%)	Reads/Total heptapeptide reads (%)
36	AβC7-70	C	A	V	W	Q	L	L	376	0.124	0.093
37	AβC7-72	C	N	V	W	Q	V	V	361	0.119	0.089
38	AβC7-75	C	K	V	W	T	V	V	335	0.11	0.083
39	AβC7-76	C	V	V	W	C	Q	P	326	0.108	0.081
40	AβC7-77	C	R	V	W	M	A	A	318	0.105	0.079
41	AβC7-78	C	R	V	W	M	T	V	316	0.104	0.078
42	AβC7-80	C	R	V	W	Q	T	R	296	0.098	0.073
43	AβC7-83	C	H	V	W	S	V	V	287	0.095	0.071
44	AβC7-85	C	Y	V	W	C	P	L	273	0.09	0.068
45	AβC7-90	C	R	V	W	E	L	L	227	0.075	0.056
46	AβC7-91	C	R	V	R	Q	E	L	220	0.073	0.054
47	AβC7-93	C	R	V	W	C	F	C	218	0.072	0.054
48	AβC7-94	C	A	V	W	C	W	P	212	0.07	0.052
49	AβC7-95	C	K	V	W	M	W	K	211	0.07	0.052
50	AβC7-97	C	R	V	W	S	V	P	207	0.068	0.051
51	AβC7-102	C	R	V	W	T	Q	V	191	0.063	0.047
52	AβC7-108	C	K	V	F	Q	V	L	173	0.057	0.043
53	AβC7-112	C	R	V	A	A	V	L	165	0.054	0.041
54	AβC7-113	C	R	V	W	C	L	P	165	0.054	0.041
55	AβC7-115	C	R	V	W	S	V	R	163	0.054	0.04
56	AβC7-118	C	V	V	W	C	T	R	161	0.053	0.04
57	AβC7-126	C	L	V	W	M	G	L	153	0.05	0.038
58	AβC7-134	C	S	V	W	Q	S	L	137	0.045	0.034
59	AβC7-137	C	R	V	W	S	P	C	134	0.044	0.033
60	AβC7-141	C	F	V	W	Q	C	R	125	0.041	0.031
61	AβC7-145	C	R	V	W	C	I	I	122	0.04	0.03
62	AβC7-146	C	R	V	W	V	P	L	121	0.04	0.03
63	AβC7-155	C	R	V	S	Q	A	L	107	0.035	0.026
64	AβC7-164	C	R	V	W	S	H	P	98	0.032	0.024
65	AβC7-166	C	R	W	W	G	G	I	97	0.032	0.024
66	AβC7-174	C	I	V	W	Q	C	L	88	0.029	0.022
67	AβC7-177	C	R	V	W	A	L	L	85	0.028	0.021
68	AβC7-179	C	R	V	S	C	P	L	84	0.028	0.021
69	AβC7-181	C	V	V	W	Q	G	L	84	0.028	0.021
70	AβC7-192	C	V	V	W	S	P	L	77	0.025	0.019
71	AβC7-204	C	R	V	W	G	A	T	71	0.023	0.018
72	AβC7-206	C	V	V	W	C	A	P	70	0.023	0.017
73	AβC7-209	C	V	V	W	S	S	L	69	0.023	0.017
74	AβC7-214	C	L	V	W	C	P	L	66	0.022	0.016
75	AβC7-220	C	R	V	W	S	G	L	60	0.02	0.015
76	AβC7-227	C	R	V	A	Q	A	L	56	0.018	0.014

Cluster I											
Number	Peptide name	Amino acid sequence							Number of reads	Reads/ Total Cluster I reads (%)	Reads/Total heptapeptide reads (%)
77	AβC7-230	C	R	V	S	A	A	L	54	0.018	0.013
78	AβC7-231	C	K	V	W	C	G	L	54	0.018	0.013
79	AβC7-232	C	V	V	T	P	V	V	54	0.018	0.013
80	AβC7-234	C	R	V	T	A	A	L	54	0.018	0.013
81	AβC7-241	C	A	V	W	Q	A	V	51	0.017	0.013
82	AβC7-245	C	R	V	W	S	A	V	50	0.016	0.012
83	AβC7-251	C	V	V	W	S	T	I	48	0.016	0.012
84	AβC7-255	C	R	V	Y	C	V	P	46	0.015	0.011
85	AβC7-262	C	R	V	W	C	A	F	44	0.015	0.011
86	AβC7-279	S	A	V	R	C	V	W	39	0.013	0.01
87	AβC7-287	C	R	V	L	C	L	G	38	0.013	0.009
88	AβC7-303	C	K	A	W	Q	S	L	34	0.011	0.008
89	AβC7-306	C	R	V	W	Q	W	V	34	0.011	0.008
90	AβC7-329	C	F	V	W	E	A	L	29	0.01	0.007
91	AβC7-331	C	R	V	W	S	E	V	28	0.009	0.007
92	AβC7-340	C	R	V	R	Q	D	L	27	0.009	0.007
93	AβC7-344	C	A	V	W	M	M	L	27	0.009	0.007
94	AβC7-349	C	R	V	W	Q	H	V	26	0.009	0.006
95	AβC7-354	C	Y	V	W	Q	S	R	26	0.009	0.006
96	AβC7-355	C	R	V	W	S	M	I	26	0.009	0.006
97	AβC7-356	C	S	V	W	C	P	L	26	0.009	0.006
98	AβC7-364	C	R	V	Y	M	E	L	25	0.008	0.006
99	AβC7-366	C	R	V	W	S	W	R	24	0.008	0.006
100	AβC7-378	C	L	V	R	Q	E	L	23	0.008	0.006
101	AβC7-396	C	K	V	W	R	L	L	21	0.007	0.005
102	AβC7-398	C	W	V	W	Q	S	L	21	0.007	0.005
103	AβC7-399	C	F	V	W	T	T	L	21	0.007	0.005
104	AβC7-401	C	V	V	W	H	V	V	21	0.007	0.005
105	AβC7-407	C	V	P	G	A	V	R	20	0.007	0.005
106	AβC7-409	C	R	V	W	R	T	L	20	0.007	0.005
107	AβC7-416	C	V	V	W	T	T	R	20	0.007	0.005
		**Sum**							**303,245**	**100**	**75.01**

Cluster II											
Number	Peptide name	Amino acid sequence							Number of reads	Reads/Total Cluster II reads (%)	Reads/Total heptapeptide reads (%)
1	AβC7-14	C	R	I	V	P	S	L	6435	32.682	1.592
2	AβC7-16	C	I	V	V	P	S	I	5871	29.817	1.452
3	AβC7-28	C	R	V	V	P	A	I	2408	12.230	0.596
4	AβC7-31	C	Q	V	V	P	S	V	2004	10.178	0.496
5	AβC7-54	C	V	V	V	P	S	I	655	3.327	0.162
6	AβC7-59	C	V	V	V	P	S	L	582	2.956	0.144
7	AβC7-81	C	L	V	V	P	S	V	291	1.478	0.072
8	AβC7-89	C	D	I	I	P	S	L	228	1.158	0.056
9	AβC7-101	C	Y	Y	V	P	S	L	198	1.006	0.049
10	AβC7-106	C	T	Y	V	P	S	L	179	0.909	0.044
11	AβC7-114	C	R	V	V	P	S	L	164	0.833	0.041
12	AβC7-160	C	K	V	V	P	T	L	101	0.513	0.025
13	AβC7-173	C	E	V	V	P	S	L	91	0.462	0.023
14	AβC7-203	C	W	T	V	G	T	I	71	0.361	0.018
15	AβC7-219	C	T	Y	V	P	S	I	63	0.320	0.016
16	AβC7-228	C	R	V	V	P	P	I	55	0.279	0.014
17	AβC7-259	C	W	V	V	G	S	I	44	0.223	0.011
18	AβC7-261	C	I	I	V	P	S	L	44	0.223	0.011
19	AβC7-276	C	W	M	V	G	S	I	41	0.208	0.010
20	AβC7-302	C	Y	W	V	P	S	L	34	0.173	0.008
21	AβC7-312	C	E	F	V	P	T	L	33	0.168	0.008
22	AβC7-335	C	M	F	V	P	T	L	28	0.142	0.007
23	AβC7-351	C	W	L	V	G	T	I	26	0.132	0.006
24	AβC7-370	C	T	V	V	P	S	V	24	0.122	0.006
25	AβC7-405	C	R	V	V	C	S	I	20	0.102	0.005
		**Sum**							**19,690**	**100**	**4.87**

Table S5. Molecular properties of the selected cyclic heptapeptides AβC7-1 and AβC7-14 compared to those of conventional drugs, oral macrocyclic (MC) drugs and nonoral MC drugs.

Property*	Conventional drugs	Oral MC drugs†	Non-oral MC drugs†	AβC7-1‡	AβC7-14‡
**MW**	≤500	600 to1200	600 to1300	870	768
**cLogP**	≤5	-2 to 6	-7 to 2	3.7	2.2
**PSA (Å²)**	≤140	180 to 320	150 to 500	285	277
**HBDs**	≤5	≤12	≤17	11	11
**HBAs**	≤10	12 to 16	9 to 20	10	11
**N_{RB}**	≤10	≤15	≤30	15	12

*Abbreviations – MC: macrocyclic; MW: molecular weight; cLogP: calculated octanol/water partition coefficient; PSA: polar surface area; HBD: hydrogen bond donor; HBA: hydrogen bond acceptor; N_{RB}: number of rotatable bonds.

†According to Villar et al. (*19*).

‡As predicted using the PerkinElmer ChemBio3D software

Table S6. Plasmids and PCR primers used in this study.

Plasmid	Encoded Protein	Marker	Origin of replication	Source
pETAβ42-GFP	Aβ42-GFP	Kan^R	ColE1	Matis et al. (*18*)
pETp53(Y220C)-GFP	p53C(Y220C)-GFP	Kan^R	ColE1	Matis et al. (*18*)
pSICLOPPS	I_C-SGGYLPPL-I_N-CBD	Cm^R	ACYC	Matis et al. (*18*)
pSICLOPPS-Cys$X_1X_2X_3X_4X_5X_6$ sub-library	I_C-Cys$X_1X_2X_3X_4X_5X_6$-I_N-CBD sub-library	Cm^R	ACYC	This work
pSICLOPPS-Ser$X_1X_2X_3X_4X_5X_6$ sub-library	I_C-Ser$X_1X_2X_3X_4X_5X_6$-I_N-CBD sub-library	Cm^R	ACYC	This work
pSICLOPPS-Thr$X_1X_2X_3X_4X_5X_6$ sub-library	I_C-Thr$X_1X_2X_3X_4X_5X_6$-I_N-CBD sub-library	Cm^R	ACYC	This work
pSICLOPPSKanR	I_C-KanR	Cm^R	ACYC	Matis et al. (*18*)
pSICLOPPS-AβC7-1	I_C-CKVWQLL-I_N-CBD	Cm^R	ACYC	This work
pSICLOPPS-AβC7-1(C1S)	I_C-SKVWQLL-I_N-CBD	Cm^R	ACYC	This work
pSICLOPPS-AβC7-1(C1T)	I_C-TKVWQLL-I_N-CBD	Cm^R	ACYC	This work
pSICLOPPS-AβC7-1(K2A)	I_C-CAVWQLL-I_N-CBD	Cm^R	ACYC	This work
pSICLOPPS-AβC7-1(V3A)	I_C-CKAWQLL-I_N-CBD	Cm^R	ACYC	This work
pSICLOPPS-AβC7-1(W4A)	I_C-CKVAQLL-I_N-CBD	Cm^R	ACYC	This work
pSICLOPPS-AβC7-1(Q5A)	I_C-CKVWALL-I_N-CBD	Cm^R	ACYC	This work
pSICLOPPS-AβC7-1(L6A)	I_C-CKVWQAL-I_N-CBD	Cm^R	ACYC	This work
pSICLOPPS-AβC7-1(L7A)	I_C-CKVWQLA-I_N-CBD	Cm^R	ACYC	This work
pSICLOPPS-AβC7-14	I_C-CRIVPSL-I_N-CBD	Cm^R	ACYC	This work
pSICLOPPS-AβC7-14(C1S)	I_C-SRIVPSL-I_N-CBD	Cm^R	ACYC	This work
pSICLOPPS-AβC7-14(C1T)	I_C-TRIVPSL-I_N-CBD	Cm^R	ACYC	This work
pSICLOPPS-AβC7-14(R2A)	I_C-CAIVPSL-I_N-CBD	Cm^R	ACYC	This work
pSICLOPPS-AβC7-14(I3A)	I_C-CRAVPSL-I_N-CBD	Cm^R	ACYC	This work

Plasmid	Encoded Protein	Marker	Origin of replication	Source
pSICLOPPS-AβC7-14(V4A)	I_C-CRIAPSL-I_N-CBD	Cm^R	ACYC	This work
pSICLOPPS-AβC7-14(P5A)	I_C-CRIVASL-I_N-CBD	Cm^R	ACYC	This work
pSICLOPPS-AβC7-14(S6A)	I_C-CRIVPAL-I_N-CBD	Cm^R	ACYC	This work
pSICLOPPS-AβC7-14(L7A)	I_C-CRIVPSA-I_N-CBD	Cm^R	ACYC	This work
pSICLOPPS-Random1	I_C-unknown peptide sequence1-I_N-CBD	Cm^R	ACYC	Matis et al. (*18*)
pSICLOPPS-Random2	I_C-unknown peptide sequence2-I_N-CBD	Cm^R	ACYC	Matis et al. (*18*)
pSICLOPPS(H24L/F26A)-AβC7-1	I_C(H24L/F26A)-CKVWQLL-I_N-CBD	Cm^R	ACYC	This work
pSICLOPPS(H24L/F26A)-AβC7-2	I_C(H24L/F26A)-CKVWMPL-I_N-CBD	Cm^R	ACYC	This work
pSICLOPPS(H24L/F26A)-AβC7-3	I_C(H24L/F26A)-CKVWTEL-I_N-CBD	Cm^R	ACYC	This work
pSICLOPPS(H24L/F26A)-AβC7-7	I_C(H24L/F26A)-CRVWQTV-I_N-CBD	Cm^R	ACYC	This work
pSICLOPPS(H24L/F26A)-AβC7-14	I_C(H24L/F26A)-CRIVPSL-I_N-CBD	Cm^R	ACYC	This work
pSICLOPPS-AβC7-2	I_C-CKVWMPL-I_N-CBD	Cm^R	ACYC	This work
pSICLOPPS-AβC7-3	I_C-CKVWTEL-I_N-CBD	Cm^R	ACYC	This work
pSICLOPPS-AβC7-7	I_C-CRVWQTV-I_N-CBD	Cm^R	ACYC	This work
pSICLOPPS-AβC7-371	I_C-CLVRSYL-I_N-CBD	Cm^R	ACYC	This work
pSICLOPPS-AβC7-405	I_C-CRVVCSI-I_N-CBD	Cm^R	ACYC	This work
pSICLOPPS-AβC7-416	I_C-CVVWTTR-I_N-CBD	Cm^R	ACYC	This work
pSICLOPPS(H24L/F26A)KanR	I_C(H24L/F26A)KanR	Cm^R	ACYC	Matis et al. (*18*)
pETAβ42	Met-Aβ42 (for ThS experiments)	Kan^R	ColE1	This work
pET-Sac-Abeta(M1-42)	Met-Aβ42 (for *in vitro* experiments)	Amp^R	ColE1	Walsh et al. (*53*)

Name	Primer sequence (5'-3')	Use
GS078	GGAATTCGCCAATGGGGCGATCGCCCACAATTGC(NNS)$_6$TGCTTAAGTTTTGGC	Degenerate forward primer for the construction of the Cys$X_1X_2X_3X_4X_5X_6$ sub-library containing a BglI site (underlined)
GS079	GGAATTCGCCAATGGGGCGATCGCCCACAATAGC(NNS)$_6$TGCTTAAGTTTTGGC	Degenerate forward primer for the construction of the Ser$X_1X_2X_3X_4X_5X_6$ sub-library containing a BglI site (underlined)
GS080	GGAATTCGCCAATGGGGCGATCGCCCACAATACC(NNS)$_6$TGCTTAAGTTTTGGC	Degenerate forward primer for the construction of the Thr$X_1X_2X_3X_4X_5X_6$ sub-library containing a BglI site (underlined)
GS035	AAAAAAAAGCTTTCATTGAAGCTGCCACAAGG	Reverse primer annealing to CBD containing a HindIII site (underlined)
GS069	AAAAAAGCCAATGGGGCGATCGCCCACAATTGC	Forward zipper primer for the construction of the Cys sub-libraries containing a BglI site (underlined)
GS070	AAAAAAGCCAATGGGGCGATCGCCCACAATAGC	Forward zipper primer for the construction of the Ser sub-libraries containing a BglI site (underlined)
GS071	AAAAAAGCCAATGGGGCGATCGCCCACAATACC	Forward zipper primer for the construction of the Thr sub-libraries containing a BglI site (underlined)
DD129	AAAAAGCCAATGGGGCGATCGCCCACAATAGCAAGGTGTGGCAGTTG	Forward primer for the construction of pSICLOPPS-AβC7-1(C1S) containing a BglI site (underlined)
DD130	AAAAAGCCAATGGGGCGATCGCCCACAATACCAAGGTGTGGCAGTTG	Forward primer for the construction of pSICLOPPS-AβC7-1(C1T) containing a BglI site (underlined)
DD131	AAAAAGCCAATGGGGCGATCGCCCACAATTGCGCGGTGTGGCAGTTGTTG	Forward primer for the construction of pSICLOPPS-AβC7-1(K2A) containing a BglI site (underlined)
DD132	AAAAAGCCAATGGGGCGATCGCCCACAATTGCAAGGCGTGGCAGTTGTTGTGC	Forward primer for the construction of pSICLOPPS-AβC7-1(V3A) containing a BglI site (underlined)
DD133	AAAAAGCCAATGGGGCGATCGCCCACAATTGCAAGGTGGCGCAGTTGTTGTGCTTAAG	Forward primer for the construction of pSICLOPPS-AβC7-1(W4A) containing a BglI site (underlined)
DD134	AAAAAGCCAATGGGGCGATCGCCCACAATTGCAAGGTGTGGGCGTTGTTGTGCTTAAG	Forward primer for the construction of pSICLOPPS-AβC7-1(Q5A) containing a BglI site (underlined)
DD135	AAAAAGCCAATGGGGCGATCGCCCACAATTGCAAGGTGTGGCAGGCGTTGTGCTTAAGTTTTG	Forward primer for the construction of pSICLOPPS-AβC7-1(L6A) containing a BglI site (underlined)
DD136	AAAAAGCCAATGGGGCGATCGCCCACAATTGCAAGGTGTGGCAGTTGGCGTGCTTAAGTTTTGG	Forward primer for the construction of pSICLOPPS-AβC7-1(L7A) containing a BglI site (underlined)
DD137	AAAAAGCCAATGGGGCGATCGCCCACAATAGCCGCATCGTCCCCAG	Forward primer for the construction of pSICLOPPS-AβC7-14(C1S) containing a BglI site (underlined)

Name	Primer sequence (5'-3')	Use
DD138	AAAAAGCCAATGGGGCGATCGCCCACAATACCCGCATCGTCCCCAG	Forward primer for the construction of pSICLOPPS-AβC7-14(C1T) containing a BglI site (underlined)
DD139	AAAAAGCCAATGGGGCGATCGCCCACAATTGCGCCATCGTCCCCAGCTTG	Forward primer for the construction of pSICLOPPS-AβC7-14(R2A) containing a BglI site (underlined)
DD140	AAAAAGCCAATGGGGCGATCGCCCACAATTGCCGCGCCGTCCCCAGCTTGTGC	Forward primer for the construction of pSICLOPPS-AβC7-14(I3A) containing a BglI site (underlined)
DD141	AAAAAGCCAATGGGGCGATCGCCCACAATTGCCGCATCGCCCCCAGCTTGTGCTTAAG	Forward primer for the construction of pSICLOPPS-AβC7-14(V4A) containing a BglI site (underlined)
DD142	AAAAAGCCAATGGGGCGATCGCCCACAATTGCCGCATCGTCGCCAGCTTGTGCTTAAG	Forward primer for the construction of pSICLOPPS-AβC7-14(P5A) containing a BglI site (underlined)
DD143	AAAAAGCCAATGGGGCGATCGCCCACAATTGCCGCATCGTCCCCGCCTTGTGCTTAAGTTTTG	Forward primer for the construction of pSICLOPPS-AβC7-14(L6A) containing a BglI site (underlined)
DD144	AAAAAGCCAATGGGGCGATCGCCCACAATTGCCGCATCGTCCCCAGCGCGTGCTTAAGTTTTGG	Forward primer for the construction of pSICLOPPS-AβC7-14(L7A) containing a BglI site (underlined)
DD175	AAAAAGCCAATGGGGCGATCGCCCACAATTGCGTGGTCTGGACGACCCGGTGCTTAAGTTTTG	Forward primer for the construction of pSICLOPPS-AβC7-416 containing a BglI site (underlined)
DD176	AAAAAGCCAATGGGGCGATCGCCCACAATTGCCGGGTGGTGTGCAGCATCTGCTTAAGTTTTG	Forward primer for the construction of pSICLOPPS-AβC7-405 containing a BglI site (underlined)
DD178	AAAAAGCCAATGGGGCGATCGCCCACAATTGCCTGGTGAGGTCCTACCTGTGCTTAAGTTTTG	Forward primer for the construction of pSICLOPPS-AβC7-371 containing a BglI site (underlined)
DD004	AAAAACCATGGATGCGGAATTTCGCCATG	Forward primer for the construction of Met-Aβ42 containing a NcoI site (underlined)
IM022	CCGCTCGAGTTACGCAATCACCACGCCGCCCAC	Reverse primer for the construction of Met-Aβ42 containing a XhoI site (underlined)
